# In vitro susceptibility testing of *Trichomonas gallinae* strains to proton pump inhibitors and nitroimidazoles

**DOI:** 10.1038/s41598-025-10668-w

**Published:** 2025-07-08

**Authors:** Ádám Kerek, Boglárka Bianka Csiki, Ábel Szabó, Máté Farkas, Andor Pitó, Ákos Jerzsele, Patrik Mag

**Affiliations:** 1 Department of Pharmacology and Toxicology, University of Veterinary Medicine Budapest, István utca 2, Budapest, H-1078 Hungary; 2 National Laboratory of Infectious Animal Diseases, Antimicrobial Resistance, Veterinary Public Health and Food Chain Safety, University of Veterinary Medicine Budapest, István utca 2, Budapest, H-1078 Hungary; 3 Department of Digital Food Science, University of Veterinary Medicine Budapest, István utca 2, Budapest, H-1078 Hungary; 4 Department of Parasitology and Zoology, University of Veterinary Medicine Budapest, István utca 2, Budapest, H-1078 Hungary; 5 HUN-REN-UVMB Climate Change: New Blood-sucking Parasites and Vector-borne Pathogens Research Group, Hungária krt. 21, Budapest, H-1143 Hungary

**Keywords:** *Trichomonas gallinae*, Proton pump inhibitors, Nitroimidazole, Pigeons, Doves, Microbiology, Zoology

## Abstract

*Trichomonas gallinae* is a unicellular obligate parasite predominantly spread among Columbiformes through the sharing of feeding and drinking stations, as well as during the feeding of squabs with crop milk. It primarily causes lesions in the pharynx and esophagus, impairing the birds’ ability to eat, drink, and even breathe, thus it is potentially fatal for infected birds. Currently, five nitroimidazole derivatives are available for treatment, which, however, are prohibited for use in food-producing livestock. The aim of this study is to explore alternative treatment options against *Trichomonas gallinae*. Parasites were collected from urban pigeons using a sterile swab from the trachea. The number of live trophozoites (per mL) was determined using a Burker chamber. Subsequently, they were treated with solutions of pantoprazole, rabeprazole dissolved in distilled water, and omeprazole, esomeprazole, and lansoprazole dissolved in ethanol. Omeprazole was found to be the most effective, achieving complete eradication at a concentration of 250 µg/mL, whereas pantoprazole and esomeprazole required a concentration of 1000 µg/mL to achieve the same result. Rabeprazole and lansoprazole were significantly less effective, requiring a concentrations of 2000 µg/mL and 4000 µg/mL for complete eradication, respectively. Overall, our findings suggest that proton pump inhibitors may serve as viable alternative treatment options for *Trichomonas gallinae* infection. The development of benzimidazole-structured compounds appears promising for the future creation and testing of new, alternative, and safer agents.

**Supplementary Information:**

The online version contains supplementary material available at 10.1038/s41598-025-10668-w.

## Introduction

*Trichomonas gallinae* (Rivolta 1878), an obligate parasite of the Trichomonadida order, primarily infects avian species, notably the feral pigeon (*Columba livia domestica*), which serves as its principal host^1^. Given the global prevalence of pigeons, *Trichomonas gallinae* has been reported in captures from diverse regions including the United States of America^2^ Africa^3^ the Middle East^4^ Europe^5^ and Australia^6^ highlighting its widespread impact. The disease it causes, trichomoniasis, predominantly spreads among avian populations through communal feeding and watering sites or during the feeding of chicks with crop milk. Predatory birds are also at risk, potentially hosting the parasite after consuming infected prey^7^.

Recent decades have seen significant research into the genetic variability of *Trichomonas* strains across different hosts, uncovering a plethora of *Trichomonas gallinae* strains. These vary in clinical manifestation from subclinical to pathogenic. Instances of concurrent infections with multiple strains in single hosts have been documented^8^. Pathologically, the disease primarily affects the oropharyngeal region, with lesions initially appearing as well-circumscribed yellow masses, which can escalate to substantial, caseous masses, obstructing nutrient intake or causing death by asphyxiation. Highly pathogenic strains may also invade the liver^9^.

The management of trichomoniasis has predominantly employed compounds from the 5-nitroimidazole group, with metronidazole and dimetridazole standing out for their effectiveness^10^. However, resistance to these drugs was noted as early as 1990, particularly in racing pigeons^11^ and has since been observed in both domesticated and wild bird populations^6,12,13^ complicating treatment efforts^14^. Furthermore, the carcinogenic potential of metronidazole^15,16^ has led to its classification as a carcinogen by the International Agency for Research on Cancer (IARC). This, coupled the resistance to nitroimidazoles, has led to prohibition of metronidazole in food-producing animals in many countries^17^.

Proton-pump inhibitors, classified within the benzimidazole derivatives known for their antiprotozoal activity, offer a potential alternative^18^. These agents disrupt critical cellular processes by binding to parasites’ β-tubulin, inhibiting microtubule polymerization^19^ impairing glucose uptake, and uncoupling oxidative phosphorylation, which lead to parasite death^20^. Additionally, proton-pump inhibitors have been suggested to inhibit the uridine nucleoside ribohydrolase enzyme, crucial for nucleotide synthesis in *Trichomonas* spp., thus presenting a viable mechanism for their antiprotozoal effect^21^. Given their structural similarities and previously demonstrated efficacy against *Trichomonas vaginalis*^18^ proton-pump inhibitors may also be effective against *Trichomonas gallinae*, including strains resistant to conventional treatments^22^.

This study aims to assess the in vitro efficacy of proton pump inhibitors on *Trichomonas gallinae* strains as an alternative therapeutic approach, in the context of increasing resistance to currently used nitroimidazoles. For comparative purposes, susceptibility data on selected nitroimidazole compounds were also included.

## Results

### Viability and reproduction

After the 24-hour incubation period, the quantification of mobile trophozoites was conducted using a Burker chamber. In order to achieve the highest accuracy, a total of 25 large squares were counted. Applying the standard cell counting formula to these observations, it was determined that the concentration reached 2,380,000 trophozoites/mL.

### Results of proton pump inhibitors treatment

The results of our analysis are visually presented to facilitate comprehensive understanding. Supplementary Figures S1-S5 display box plots that delineate the distribution of values obtained for each active substance, including measures such as the mean, median, quartiles, and standard deviation. This graphical representation offers an intuitive overview of the data’s spread and central tendency across different treatments.

On the other hand *Supplementary Figures S6-S10* illustrate the impact of various concentrations of each active substance on the percentage reduction of parasite numbers. These figures provide a detailed depiction of the efficacy of each concentration, allowing for direct comparisons and a clearer understanding of how different concentration influence parasite control. Through these supplementary figures, readers can gain insights into the statistical nuances and the practical implications of our findings, underscoring the potential of the active substances in question for managing parasite infections effectively.

**Fig. 1:**
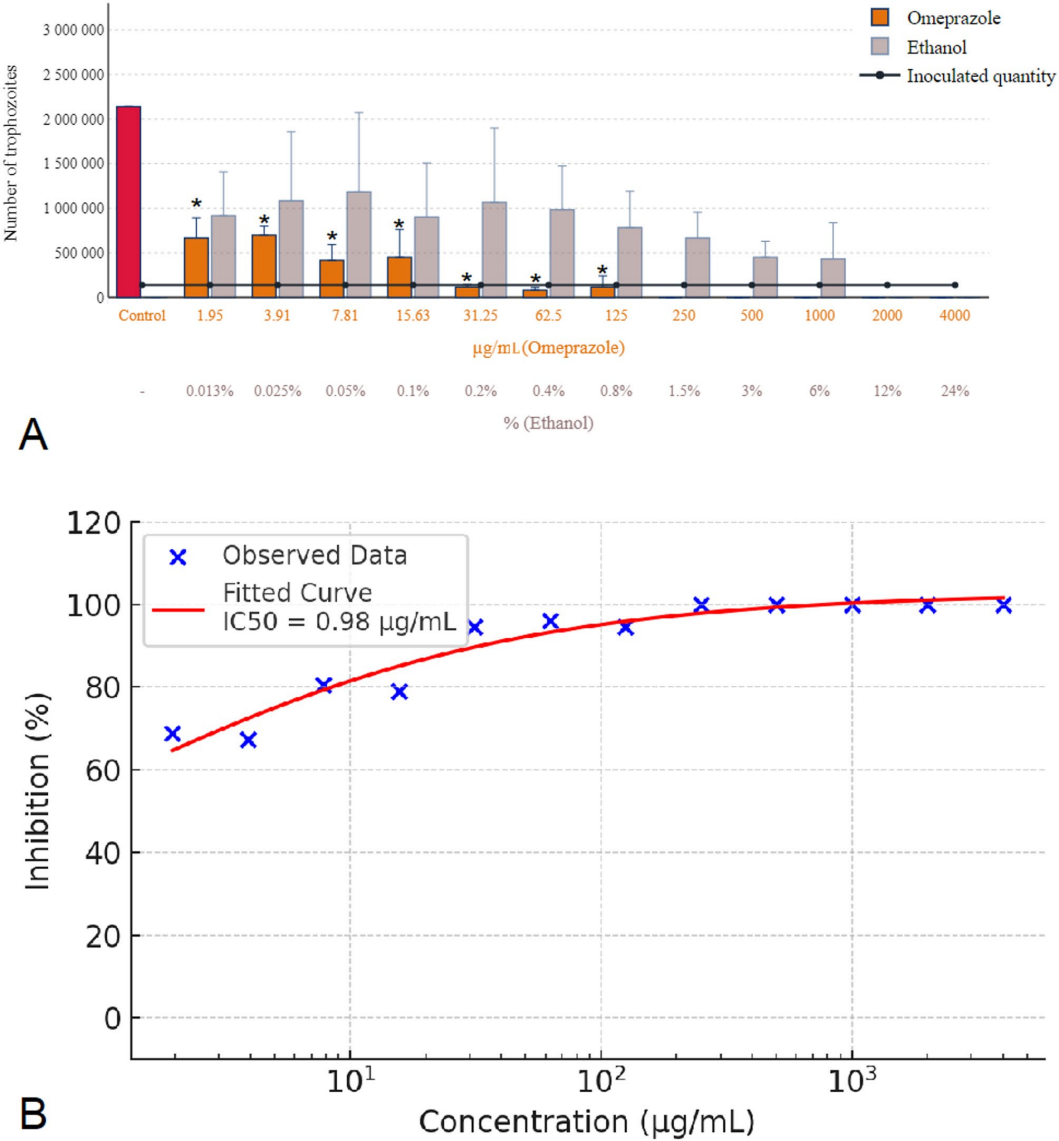
Effect of omeprazole and ethanol solvent on *Trichomonas gallinae* trophozoite counts and concentration-response curve. (**A**) Trophozoite counts after 24-hour incubation with omeprazole (1.95–4000 µg/mL) and ethanol solvent controls (0.013-24%). Each data point represents the mean of three independent experiments (*n* = 3), each performed on separate days using distinct parasite cultures. Within each experiment, triplicate wells (technical replicates) were analyzed and averaged to obtain one value per concentration. (**B**) Concentration-response curve of omeprazole based on these biological replicates. A four-parameter logistic (4PL) model was fitted to the data (red sigmoidal curve), and IC₅₀ values were calculated. Concentrations are shown on a logarithmic scale. Error bars represent standard deviation (SD) of the three independent experiments. Asterisks indicate statistical significance versus control (* *p* < 0.05).

In the comparative analysis of proton pump inhibitors, omeprazole demonstrated exceptional efficacy, achieving complete eradication of *Trichomonas gallinae* at a concentration of 250 µg/mL, as shown in Fig. 1A. Concurrently, the ethanol used as a solvent in this experiment exhibited parasiticidal activity at a concentration of 12%, aligning with the corresponding dilution level. This activity was significantly pronounced (*p* < 0.001) at concentrations up to 3%, relative to the parasite numbers, indicating a notable impact of ethanol on parasite viability. However, at a reduced concentration of 1.5%, the same concentration presents in the 250 µg/mL omeprazole solution, ethanol did not significantly affect parasite numbers, underscoring that the observed eradication was attributable to the action of omeprazole rather than the solvent. Figure 1B shows the concentration-response curve of omeprazole, indicating an estimated IC₅₀ of 0.98 µg/mL.

**Fig. 2:**
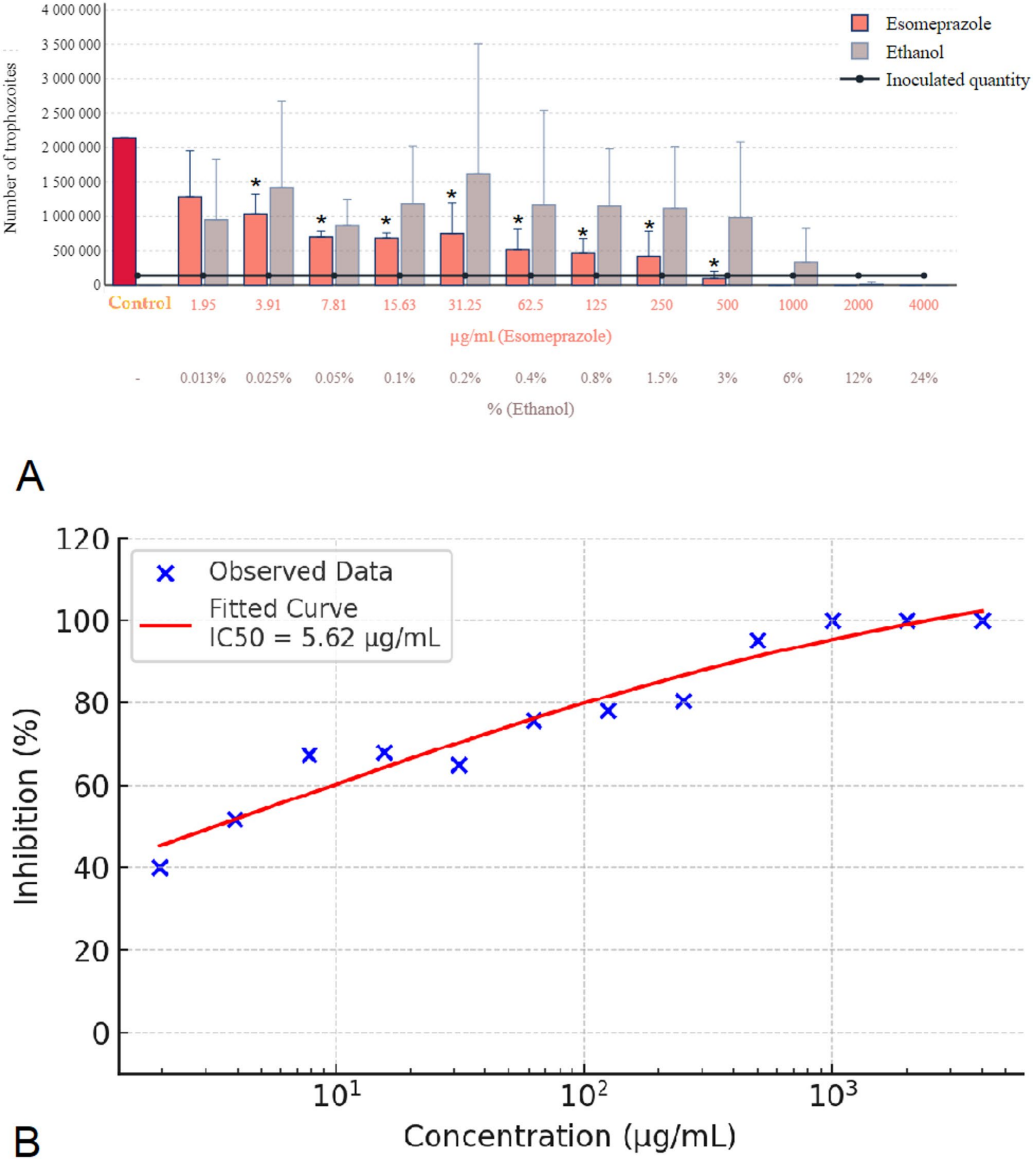
Effect of esomeprazole and ethanol solvent on *Trichomonas gallinae* trophozoite counts and concentration-response curve. (**A**) Trophozoite counts after 24-hour incubation with esomeprazole (1.95–4000 µg/mL) and ethanol solvent controls. Each data point represents the mean of three independent experiments (*n* = 3), each conducted on separate days using independently prepared parasite cultures. In each experiment, triplicate wells (technical replicates) were used and their average was calculated to obtain a single data point per concentration. (**B**) Concentration-response curve of esomeprazole based on the means of the three biological replicates. A four-parameter logistic (4PL) model was fitted to the data (red sigmoidal curve), and IC₅₀ values were calculated. Concentrations are shown on a logarithmic scale. Error bars represent standard deviation (SD) across the three independent experiments. Asterisks indicate statistical significance versus control (* *p* < 0.05).

For esomeprazole, achieving complete elimination of *Trichomonas gallinae* necessitated a concentration of 1000 µg/mL, as depicted in Fig. 2A. Ethanol, serving as the solvent, exhibited a parasiticidal effect at a 24% concentration. This effect was significant at 12% concentration, where it notably reduced parasite numbers, and remained observable albeit diminished at 6%. However, the parasiticidal action of ethanol was not evident at concentrations below 3%. Given these results, the possibility of a synergistic interaction between esomeprazole and ethanol, contributing to the parasiticide effect, cannot be dismissed. The overlapping parasiticidal effects of the active substance and the solvent at higher concentrations suggest a potential combined action that warrants further investigation. Figure 2B shows the concentration-response curve of esomeprazole, indicating an estimated IC₅₀ of 5.62 µg/mL.

**Fig. 3:**
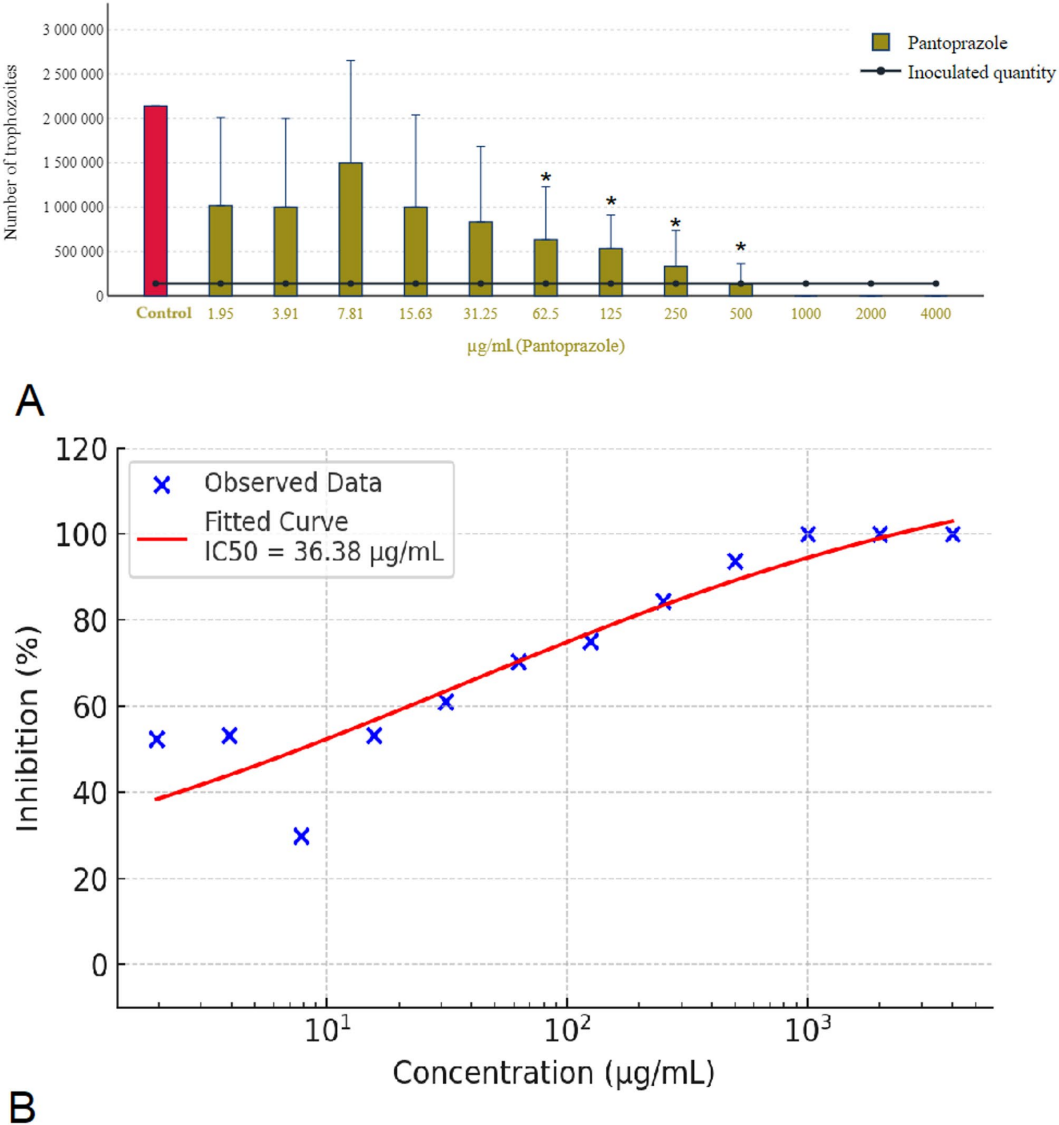
Effect of pantoprazole on *Trichomonas gallinae* trophozoite counts and concentration-response curve. (**A**) Trophozoite counts after 24-hour incubation with pantoprazole (1.95–4000 µg/mL). Each data point represents the mean of three independent experiments (*n* = 3), each performed on different days using freshly cultured parasite populations. Within each experiment, three technical replicates were used, and their average was calculated to yield one value per concentration. (**B**) Concentration-response curve of pantoprazole calculated from these biological replicate means. A four-parameter logistic (4PL) model was fitted to the data (red sigmoidal curve), and IC₅₀ values were calculated. Concentrations are shown on a logarithmic scale. Error bars represent standard deviation (SD) of the three independent experiments. Asterisks indicate statistical significance versus control (*p < 0.05).

Pantoprazole, as illustrated in Fig. 3A, demonstrated its solubility in water, which effectively eliminated concerns regarding solvent interference in its antiparasitic activity. To achieve complete eradication of the parasites, a concentration of 1000 µg/mL was necessary. Interestingly, at a lower concentration of 500 µg/mL, pantoprazole exhibited a significant anti-replication effect on the parasites. This observation suggests that while higher concentrations are required for total eradication, pantoprazole begins exerting its inhibitory effects on parasite replication at lower concentration. Figure 3B shows the concentration-response curve of pantoprazole, indicating an estimated IC₅₀ of 36.38 µg/mL.

**Fig. 4:**
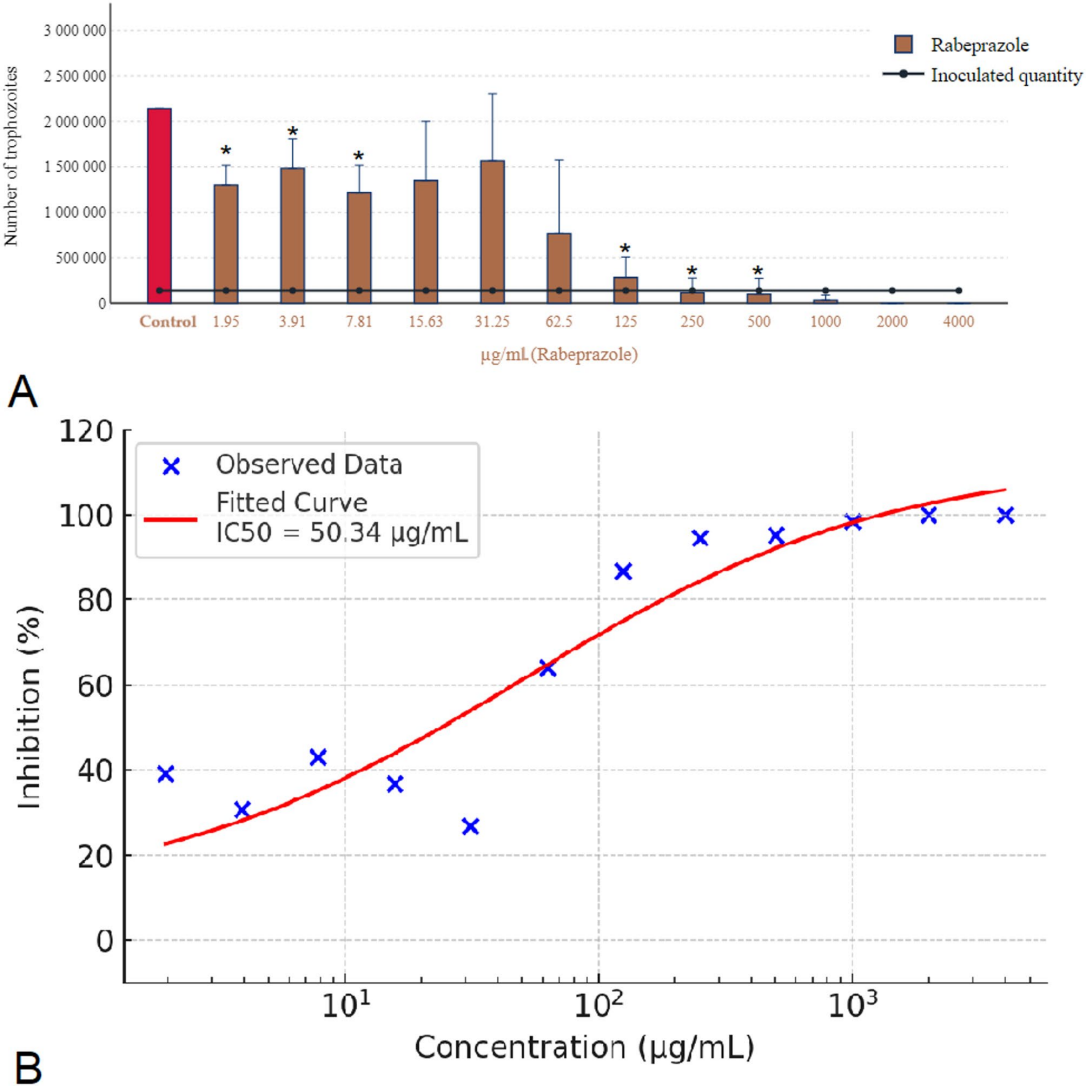
Effect of rabeprazole on *Trichomonas gallinae* trophozoite counts and concentration-response curve. (**A**) Trophozoite counts after 24-hour incubation with rabeprazole (1.95–4000 µg/mL). Water was used as solvent control. Each data point represents the mean of three independent experiments (*n* = 3), each carried out on separate days using distinct parasite cultures. Within each experiment, triplicate wells (technical replicates) were used, and the values averaged to yield one representative value per concentration. (**B**) Concentration-response curve of rabeprazole derived from these biological replicate means. A four-parameter logistic (4PL) model was fitted to the data (red sigmoidal curve), and IC₅₀ values were calculated. Concentrations are shown on a logarithmic scale. Error bars represent standard deviation (SD) across the three independent experiments. Asterisks indicate statistical significance versus control (*p < 0.05).

Rabeprazole demonstrated a comparatively lower efficacy against *Trichomonas gallinae* than pantoprazole, as detailed in Fig. 4A. For rabeprazole, a concentration of 2000 µg/mL was necessary to achieve complete eradication of the parasites. However, its capacity to inhibit parasite reproduction was evident at concentrations as low as 500 µg/mL. Given that the solvent used for rabeprazole was water, no solvent-related effects were observed on the parasite’s viability or reproduction. This distinction underlines rabeprazole’s inherent antiparasitic activity, albeit at higher required concentrations for full efficacy compared to pantoprazole. Figure 4B shows the concentration-response curve of rabeprazole, indicating an estimated IC₅₀ of 50.34 µg/mL.

**Fig. 5:**
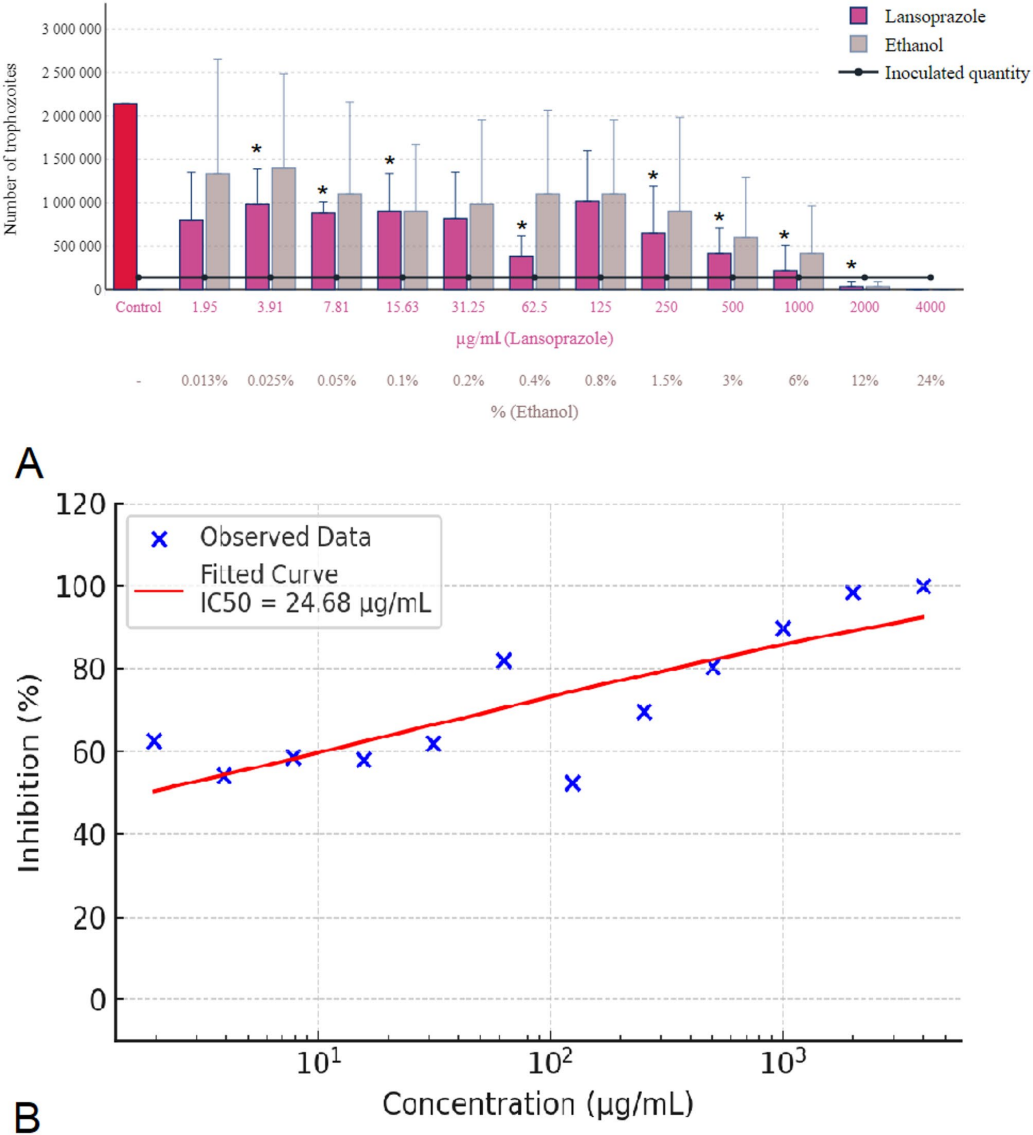
Effect of lansoprazole on *Trichomonas gallinae* trophozoite counts and concentration-response curve. (**A**) Trophozoite counts after 24-hour incubation with lansoprazole (1.95–4000 µg/mL) and ethanol solvent controls. Each data point represents the mean of three independent experiments (*n* = 3), performed on separate days using distinct parasite cultures. Within each experiment, triplicate wells (technical replicates) were analyzed, and their average was used to obtain a single value per concentration. (**B**) Concentration-response curve of lansoprazole constructed from the means of the three biological replicates. A four-parameter logistic (4PL) model was fitted to the data (red sigmoidal curve), and IC₅₀ values were calculated. Concentrations are shown on a logarithmic scale. Error bars represent standard deviation (SD) across the three independent experiments. Asterisks indicate statistical significance versus control (* *p* < 0.05).

Lansoprazole demonstrated its capacity to completely eradicate *Trichomonas gallinae* at a concentration of 4000 µg/mL, a potency mirrored by the corresponding 24% concentration of ethanol, as depicted in Fig. 5A. At a reduced concentration of 2000 µg/mL, both lansoprazole and the equivalent 12% ethanol concentration significantly reduced parasite numbers and inhibited their reproduction to a similar degree. However, the efficacy of lansoprazole in inhibiting parasite reproduction began to wane at concentrations lower than 1000 µg/mL. This reduction in inhibitory effect was noted up to a concentration threshold of 125 µg/mL, beyond which a steady increase in parasite numbers was observed, indicating the parasites were able to reproduce under these conditions. Figure 5B shows the concentration-response curve of lansoprazole, indicating an estimated IC₅₀ of 24.68 µg/mL.

**Fig. 6:**
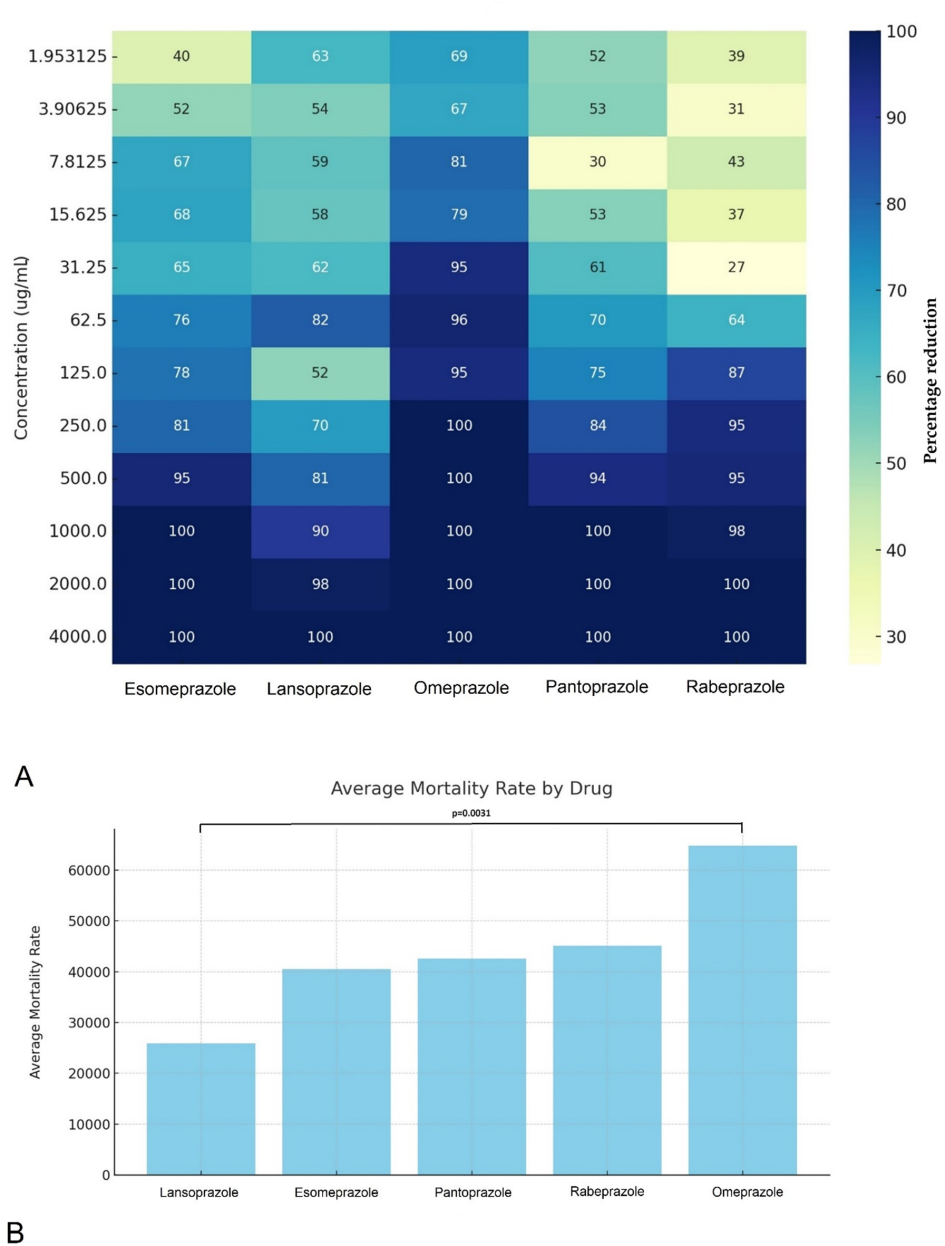
Comparison of the antiparasitic efficacy of proton pump inhibitors. (**A**) Percentage reduction in trophozoite counts at different concentrations of each active substance, measured after 24-hour incubation. (**B**) Average mortality rates attributed to each active substance. Each data point represents the mean of three independent experiments (*n* = 3), each performed on different days with separately prepared parasite cultures. For each experiment, triplicate technical replicates were used, and their average was taken to yield a single value per concentration per experiment. Statistical comparison of efficacy was performed using the Mann-Whitney U test based on the three biological replicates per treatment group. Error bars represent standard deviation (SD) across the three independent experiments.

We visualized the relationship between the concentration of each active substance and its corresponding percentage reduction in parasite number using a heat map (Fig. 6A). This approach allows for an at-a-glance understanding of concentration-response effects across the range of substances tested, highlighting the concentrations that are most effective in reducing parasite numbers.

Subsequently, we charted the average mortality rate attributable to each active substance in Fig. 6B, enabling a comparative analysis of efficacy among the different groups. Our statistical analysis revealed significant differences in the effectiveness of lansoprazole compared to omeprazole, as indicated by a p-value of 0.0031. However, for all other comparisons between active agents, the differences were not statistically significant, with p-values exceeding the 0.05 threshold. This finding underscores the distinct antiparasitic potential of lansoprazole and omeprazole, suggesting that these two compounds may warrant further investigation for their unique efficacy profiles against parasite infections.

In summary, the active substances evaluated in this study suggest that proton pump inhibitors possess potential efficacy in the treatment of *Trichomonas gallinae* infections. Among the drugs tested, omeprazole demonstrated the most significant efficacy at a concentration of 250 µg/mL, with a clear allowance for any contributory effects from the ethanolic solvent. Somewhat less effective than omeprazole were pantoprazole and esomeprazole, both requiring a concentration of 1000 µg/mL for effective parasitic control, without any observable influence from the solvent. Conversely, rabeprazole and lansoprazole, required concentrations of 2000 µg/mL and 4000 µg/mL respectively. This hierarchy of efficacy highlights the varied potential of proton pump inhibitors in the treatment of *Trichomonas gallinae*, underscoring omeprazole’s prominence due to its efficacy at lower concentrations and clear non-reliance on solvent effects.

Exact p-values for each concentration of the tested proton pump inhibitors compared to the control are provided in Supplementary Table 1.

### Results of nitroimidazoles treatment

Supplementary Figures S11-S14 show box plots that delineate the distribution of values obtained for ronidazole, metronidazole, tinidazole and secnidazole, including measures such as the mean, median, quartiles, and standard deviation.

**Fig. 7:**
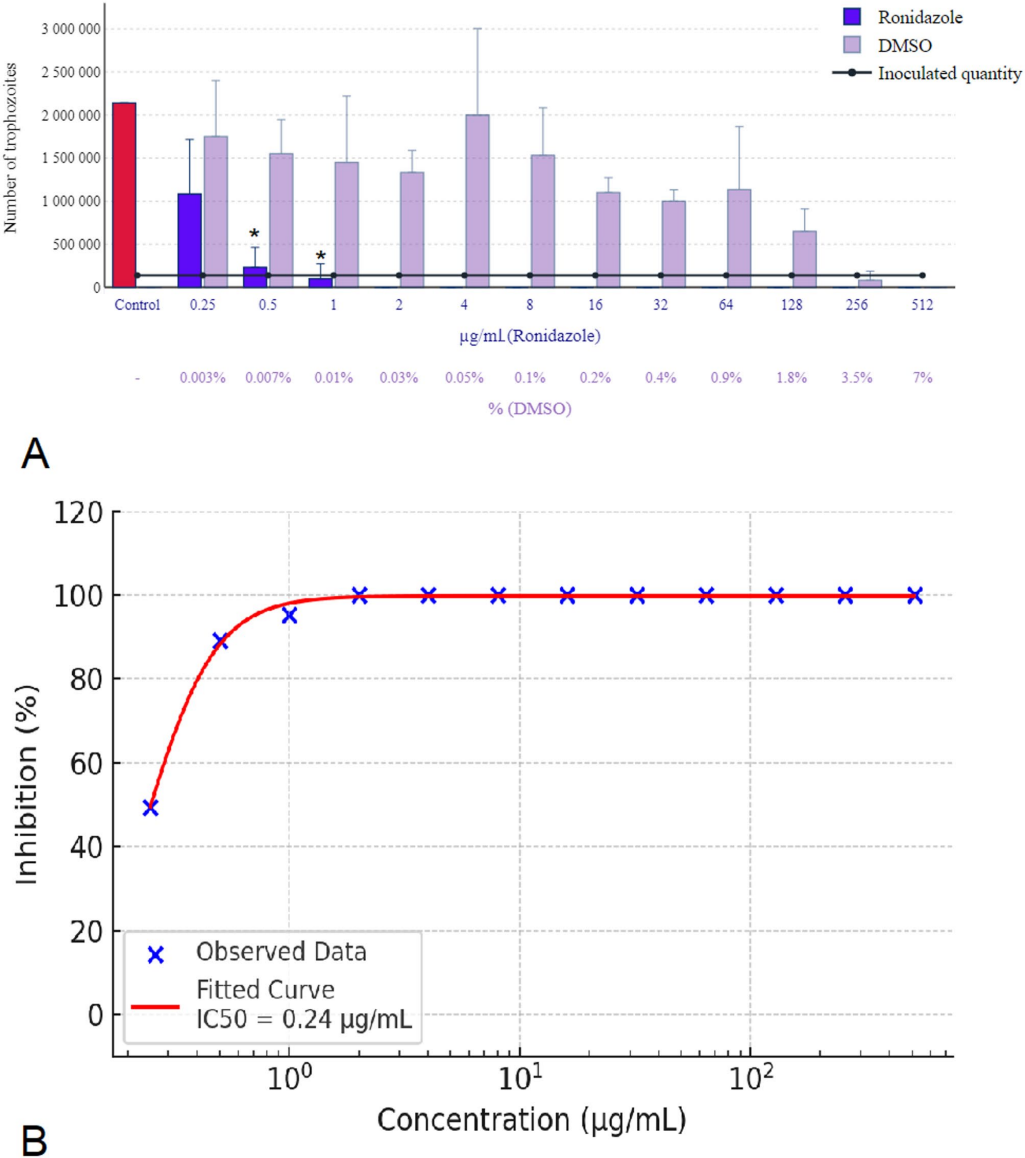
Effect of ronidazole on *Trichomonas gallinae* trophozoite counts and concentration-response curve. (**A**) Trophozoite counts after 24-hour incubation with ronidazole at various concentrations (0.25–512 µg/mL). (**B**) Concentration-response curve of ronidazole based on three independent experiments (*n* = 3), each conducted on separate days with freshly prepared parasite cultures. Within each experiment, triplicate wells (technical replicates) were used, and their average was taken to represent each concentration. A four-parameter logistic (4PL) model was fitted to the data (red sigmoidal curve), and IC₅₀ values were calculated. Concentrations are shown on a logarithmic scale. Error bars represent standard deviation (SD) across the three independent experiments. Asterisks indicate statistical significance versus control (*p < 0.05).

For all nitroimidazole active substances used as positive controls, the effective concentration of DMSO used as the solvent was below 0.05% (v/v), thereby negating its potential influence. Among these, ronidazole was the most effective, with a minimum parasiticidal concentration of 2 µg/mL achieving complete eradication of parasites as depicted in Fig. 7A. This figure also illustrates that DMSO inhibited parasite growth at a concentration of only 3.5% and exhibited parasiticidal effects at 7%. Figure 7B shows the concentration-response curve of ronidazole, indicating an estimated IC₅₀ of 0.24 µg/mL.

**Fig. 8:**
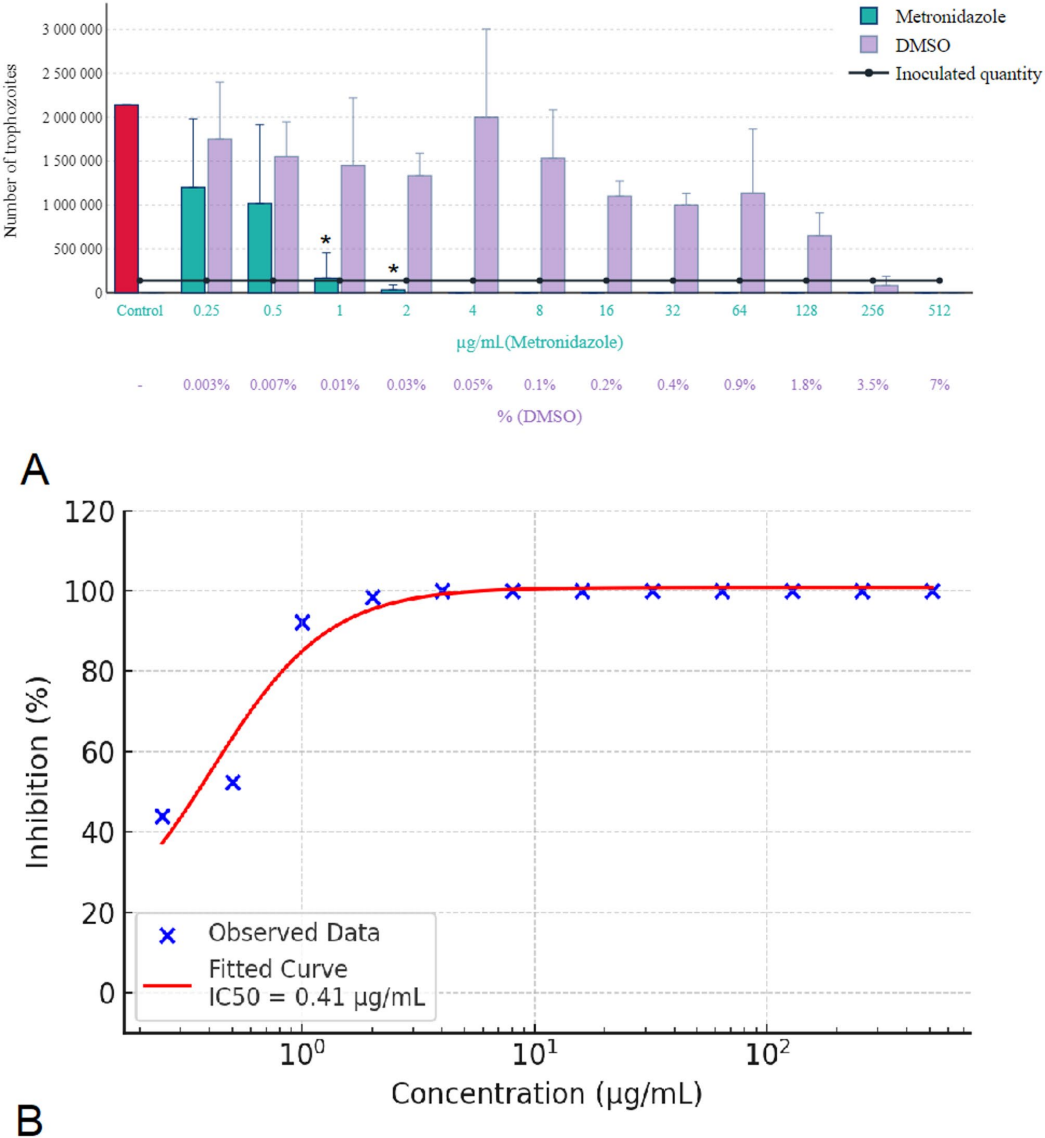
Effect of metronidazole on *Trichomonas gallinae* trophozoite counts and concentration-response curve. (**A**) Trophozoite counts after 24-hour incubation with metronidazole at concentrations of 0.25–512 µg/mL. (**B**) Concentration-response curve of metronidazole calculated from three independent experiments (*n* = 3), each performed on separate days using independent parasite cultures. In each experiment, three technical replicates were included per concentration, and their meaning was used for analysis. A four-parameter logistic (4PL) model was fitted to the data (red sigmoidal curve), and IC₅₀ values were calculated. Concentrations are shown on a logarithmic scale. Error bars represent standard deviation (SD) across the three biological replicates. Asterisks indicate statistical significance versus control (* *p* < 0.05).

**Fig. 9:**
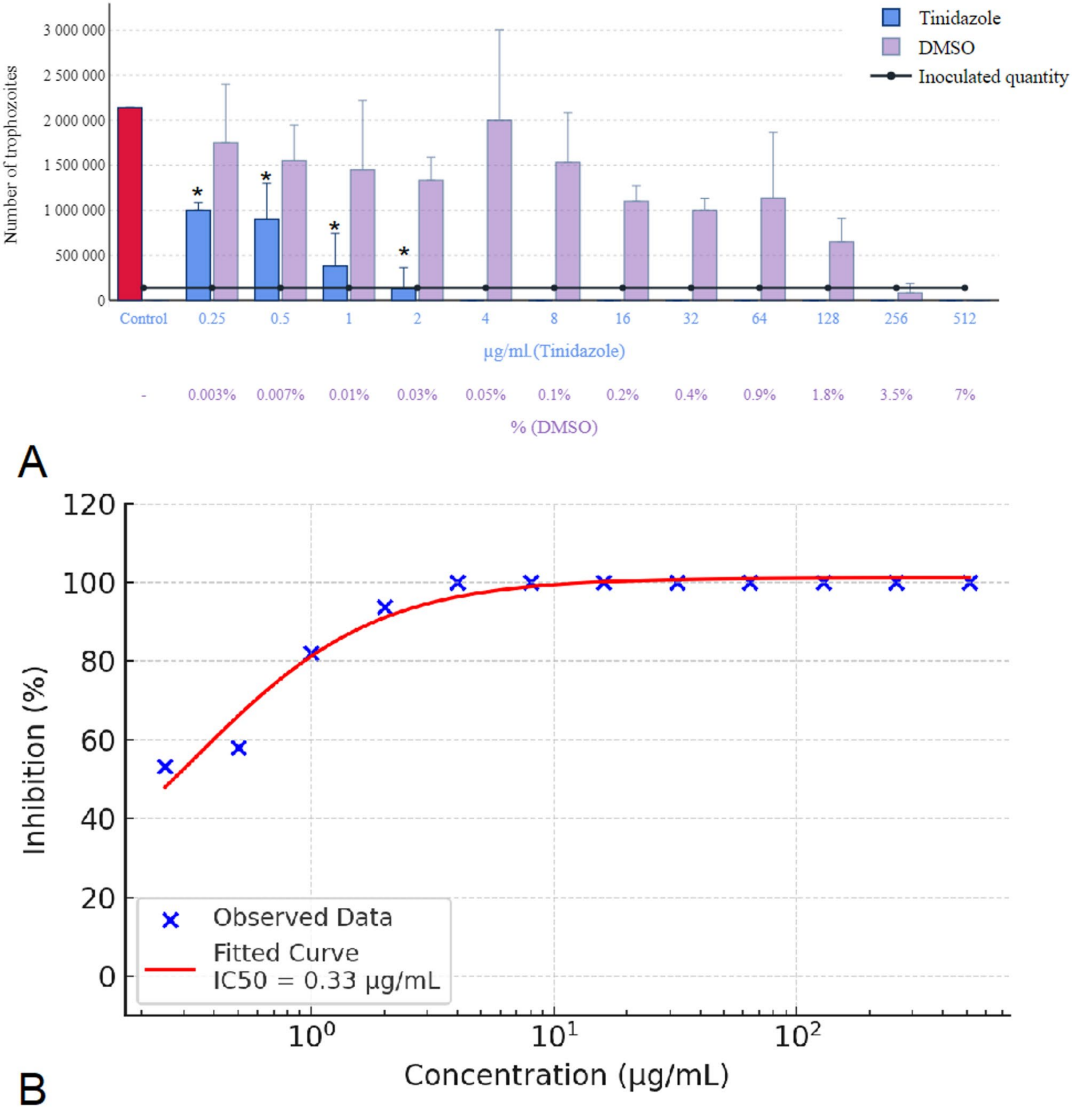
Effect of tinidazole on *Trichomonas gallinae* trophozoite counts and concentration-response curve. (**A**) Trophozoite counts after 24-hour incubation with tinidazole at concentrations of 0.25–512 µg/mL. (**B**) Concentration-response curve of tinidazole based on three independent experiments (*n* = 3), each conducted on different days with separately cultured parasite isolates. For each experiment, triplicate technical replicates were used, and their average was used to generate a single value per concentration. A four-parameter logistic (4PL) model was fitted to the data (red sigmoidal curve), and IC₅₀ values were calculated. Concentrations are shown on a logarithmic scale. Error bars represent standard deviation (SD) across the three independent experiments. Asterisks indicate statistical significance versus control (*p < 0.05).

**Fig. 10:**
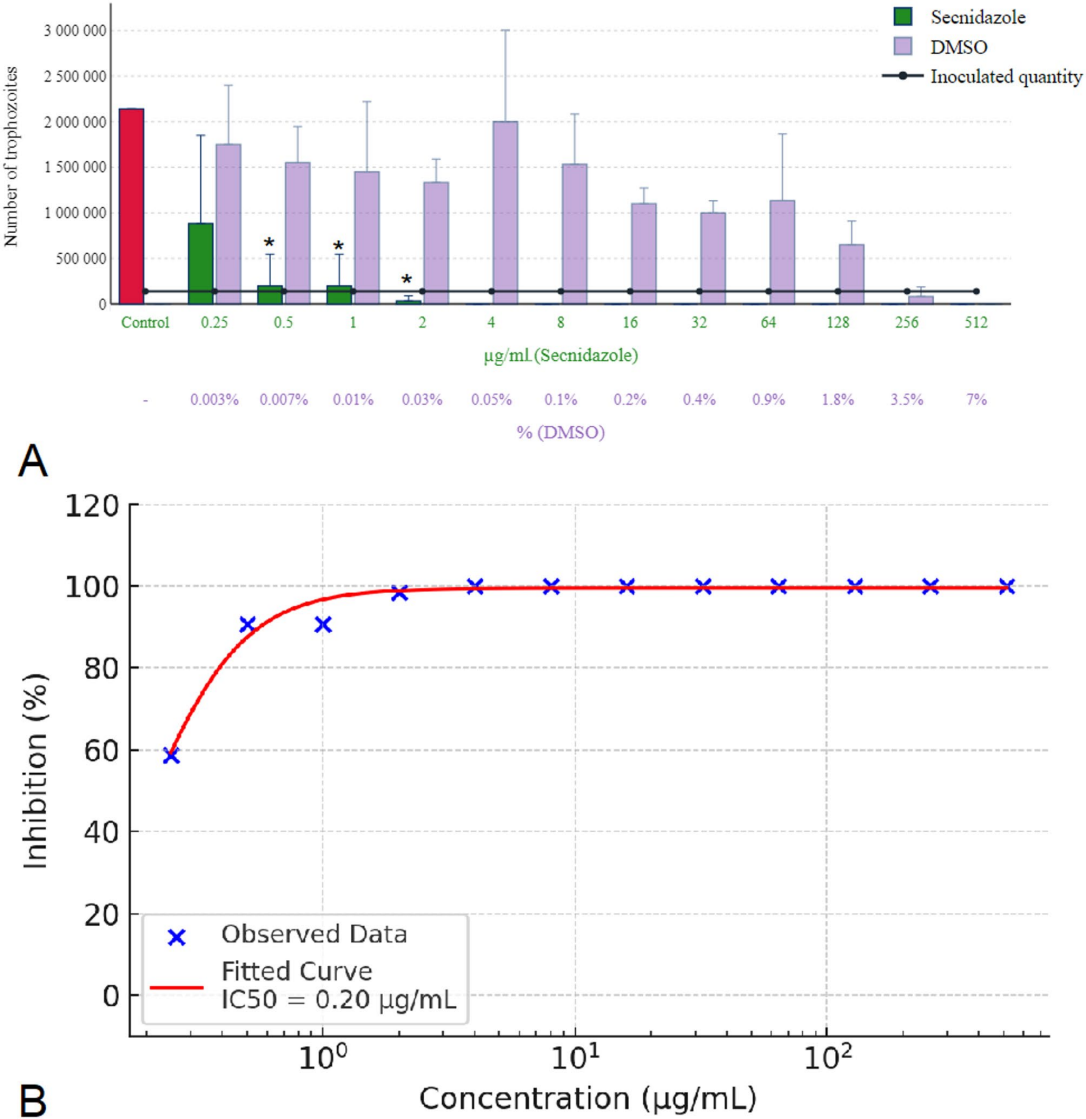
Effect of secnidazole on *Trichomonas gallinae* trophozoite counts and concentration-response curve. (**A**) Trophozoite counts after 24-hour incubation with secnidazole at concentrations of 0.25–512 µg/mL. (**B**) Concentration-response curve of secnidazole based on three independent experiments (*n* = 3), each performed on different days using independently cultured parasite populations. Within each experiment, triplicate technical replicates were averaged to generate a single value per concentration. A four-parameter logistic (4PL) model was fitted to the data (red sigmoidal curve), and IC₅₀ values were calculated. Concentrations are shown on a logarithmic scale. Error bars represent standard deviation (SD) across the three independent experiments. Asterisks indicate statistical significance versus control (*p < 0.05).

The results for metronidazole, tinidazole, and secnidazole (Figs. 8 and 9 A) were consistent, with all requiring a concentration of 4 µg/mL for complete eradication. In each case, the solvent inhibited parasite growth at concentrations above 3.5% and was parasiticidal at concentrations above 7%. Below a threshold of 1.8%, however, DMSO had no effect on parasite growth. The concentration-response curve of metronidazole is presented in Fig. 8B, demonstrating a concentration-dependent inhibition with an estimated IC₅₀ of 0.41 µg/mL. Similarly, Fig. 9B illustrates the sigmoidal inhibition profile of tinidazole, with an IC₅₀ value of 0.33 µg/mL. Secnidazole exhibited the highest potency among the nitroimidazoles tested, with a steep concentration-response curve and an estimated IC₅₀ of 0.20 µg/mL, as shown in Fig. 10B.

In summary, ronidazole was the most potent nitroimidazole active substance (2 µg/mL), followed by metronidazole, tinidazole, and secnidazole, which all demonstrated equal efficacy at 4 µg/mL. These are notable for their potential to either partially or fully substitute for traditional antibiotics.

Detailed statistical comparisons between nitroimidazole-treated groups and controls at each tested concentration are presented in Supplementary Table 2.

## Discussion

In assessing *Trichomonas gallinae*, a notable scarcity of comparative literature exists, with the bulk of research focusing on *Trichomonas vaginalis*, a protozoan within the same genus. Given the escalating challenge of antimicrobial resistance globally in the 21st century, the exploration of natural alternatives to antibiotics has become critically important. Among such alternatives, plant extracts and essential oils^23–27^ propolis^28–30^ probiotics^31,32^ and antimicrobial peptides^33^. Besides all this, adhering to the appropriate biosecurity measures is also of paramount importance^34^.

The study included three *Trichomonas gallinae* strains, each isolated from different individual pigeons. While the in vitro findings appear promising, the limited number of strains means that the data should be considered primarily exploring. Further investigations involving a larger number of isolates and genetically diverse populations are warranted to confirm the generalizability of the observed effects.

Our study reveals the promising application of proton pump inhibitors as viable alternatives to antibiotics, as evidenced by in vitro testing. Specifically, omeprazole exhibited exceptional efficacy, completely eradicating parasites at a concentration of 250 µg/mL within 24 h, while also demonstrating an inhibitory effect on *Trichomonas gallinae* reproduction at concentrations as low as 31.25 µg/mL. This finding resonates with Pérez-Villanueva et al.’s observation of total eradication of *Trichomonas vaginalis* by proton pump inhibitors (omeprazole, lansoprazole, rabeprazole and pantoprazole) at 352 µg/mL^18^. In contrast, pantoprazole required a 1000 µg/mL concentration for full eradication, though Gökmen et al. reported its effectiveness against *Trichomonas vaginalis* strains at as low as 200 µg/mL^22^ a finding also supported by Pérez-Villanueva et al.^18^. For rabeprazole, our study indicated a requisite 2000 µg/mL for complete parasite eradication, a contrast to Pérez-Villanueva et al.’s report of full eradication at 300 µg/mL^18^. For *Trichomonas vaginalis*. Esomeprazole necessitated a 1000 µg/mL concentration for total protozoan elimination, in alignment with Gökmen et al.’s findings of efficacy at 400 µg/mL against *Trichomonas vaginalis*^22^. Lansoprazole proved least effective, requiring 4000 µg/mL for complete eradication, whereas Pérez-Villanueva et al. identified a 300 µg/mL concentration as effective against *Trichomonas vaginalis*^18^.

Although the in vitro minimum antiparasitic concentration of omeprazole (250 µg/mL) exceeds the plasma levels reported in mammals^35,36^ the oropharyngeal localization of *Trichomonas gallinae* provides a unique opportunity for local administration, whereby the compound can be delivered directly to the target site at high concentrations without inducing systemic toxicity.

The variance in efficacy we observed among different proton-pump inhibitors can be attributed to their distinct molecular structures, influenced by the substitution groups on the benzimidazole ring^18^a core component of these benzimidazole-derived heterocyclic molecules^37^. This suggests the potential for in silico modeling to refine each active substance group, leveraging preliminary screening and in vitro sensitivity outcomes. Notably, lansoprazole has undergone drug target prediction studies for parasites, demonstrating its ability to bind to calcium transporting ATPases. Docking studies of all its enantiomers have shown very stable complexes with all isoforms of the P-type Ca^2+^ ATPase This suggests that disrupting these critical energetic functions could impair the calcium homeostasis of parasites and thereby explain the antiparasitic efficacy of this compound class^38^. Modulators of calcium channels are known to enhance the effectiveness of antiparasitic agents and represent promising targets in addressing the growing challenge of drug resistance^39^. Furthermore, P-type ATPases play a vital role in maintaining lipid membrane asymmetry and cellular ion homeostasis by transporting phospholipids and ions against their concentration gradients^40^ and they may also contribute to antiparasitic activity through the reduction of intracellular sulfoxide^41^. In terms of solubilization, proton pump inhibitors like omeprazole, esomeprazole, and lansoprazole require ethanol, whereas pantoprazole and rabeprazole were fully water-soluble. Ethanol concentrations exceeding 6% were found to completely eliminate parasites within 24 h, and even concentrations above 3% resulted in a significant reduction in parasite numbers (*p* < 0.001). For the nitroimidazole active substances, the influence of DMSO used as the solvent was negligible, even at the very low effective concentrations employed.

Among the tested compounds, notable differences in antiparasitic potency were observed based on their IC₅₀ values, as illustrated in Figs. 2–20. Within the group of proton pump inhibitors (PPIs), omeprazole exhibited the highest efficacy, with an IC₅₀ of 0.98 µg/mL (Fig. 2), followed by esomeprazole (5.62 µg/mL; Fig. 4), lansoprazole (24.68 µg/mL; Fig. 10), pantoprazole (36.38 µg/mL; Fig. 6), and rabeprazole, which showed the lowest potency among the PPIs with an IC₅₀ of 50.34 µg/mL (Fig. 8). These findings suggest that subtle structural or physicochemical differences among PPIs may significantly influence their antiparasitic activity.

In comparison, the nitroimidazole-class compounds demonstrated markedly greater potency. Secnidazole exhibited the strongest activity (IC₅₀ = 0.20 µg/mL; Fig. 10), followed by ronidazole (0.24 µg/mL; Fig. 7), tinidazole (0.33 µg/mL; Fig. 9), and metronidazole (0.41 µg/mL; Fig. 8). The low IC₅₀ values and steep sigmoidal concentration-response profiles observed in this group underscore their well-documented efficacy against protozoa. Notably, secnidazole displayed the most potent inhibition overall, highlighting its potential as a lead candidate for further development or repositioning in antiparasitic therapy.

The notably lower effective concentrations required for nitroimidazoles suggest that these compounds may present a lower toxicological risk in practical applications. Although higher concentrations of PPIs are generally well-tolerated, species-specific toxicological studies are essential to evaluate their safety in veterinary applications. For instance, in mice, the oral LD_50_ for omeprazole and pantoprazole is reported as 4000 µg/kg^42^. However, in rabbits, a 28-day toxicity study demonstrated that doses of esomeprazole up to 40 mg/animal and 120 mg/animal were safe^43^. Similarly, long-term administration of lansoprazole in rats, at a dose of 5 mg/kg/day, did not produce any lesions^44^. Furthermore, rabeprazole was found to be safe in dogs at doses up to 30 mg/kg^45^. Given the primary oropharyngeal cavity location of *Trichomonas gallinae*, topical application of these active substances could mitigate the risk of systemic toxicity. However, if oral administration is considered, higher concentration would be required to achieve adequate tissue concentrations, necessitating careful concentration optimization.

One limitation of the present study is the lack of a recovery assay to assess the potential for parasite regrowth following drug withdrawal. Such assays, involving re-incubation in drug-free medium after treatment-induced inhibition, would provide additional insights into whether the observed antiparasitic effects are reversible or sustained.

Investigating the potential combinatory effects of PPIs and nitroimidazoles — for example through checkerboard assays — could be of considerable interest for future studies, as synergistic interactions may allow for the development of lower- concentration combination therapies against *Trichomonas gallinae*.

## Materials and methods

### Origin of the parasites and substances tested

*Trichomonas gallinae* protozoan parasites were isolated from the tracheae of urban pigeons in Budapest, utilizing a sterile Amies swab. This collection was performed by an authorized technician, employing samplers comprised of standard aluminum rods without carbon components, provided by Biolab Zrt., Budapest, Hungary. Following collection, the samples were immediately transported to the Microbiology Laboratory within the Department of Pharmacology and Toxicology. For transport, a specialized culture medium was employed, maintained at a temperature of 37 °C, which was also used for the subsequent maintenance and propagation of the parasites. The chemical reagents and standard substances used throughout the study were sourced from Merck KGaA, Darmstadt, Germany, ensuring consistency and reliability in the experimental procedures.

All animal sampling procedures were reviewed by the Animal Welfare Committee of the University of Veterinary Medicine Budapest, which confirmed that the study entitled *“In vitro Susceptibility Testing of Trichomonas gallinae Strains to Proton Pump Inhibitors and Nitroimidazoles”* does not constitute an animal experiment under Hungarian law (Act XXVIII of 1998 on Animal Protection and Government Decree No. 40/2013 on animal experimentation). A Certificate of Exemption was issued by the committee. No invasive procedures were performed on animals. Tracheal swab samples were obtained during routine diagnostic procedures by a licensed veterinarian holding specific authorization for bird capture and sampling. All methods were conducted in full compliance with national legislation and institutional animal welfare guidelines. This study is reported in accordance with the ARRIVE guidelines 2.0 (https://arriveguidelines.org).

### Maintenance and reproduction of protozoa

The culture medium utilized for both the initial processing of incoming swab samples and the subsequent reproduction of protozoa was prepared to support optimal growth conditions. This specialized broth consisted of:

- *Trichomonas* cysteine peptone liver infusion medium (CPLM): A foundational component of the medium, occupying a volume of 425 mL, specifically designed to foster the growth and maintenance of *Trichomonas* species.
- Trichomonas selective supplement: One vial of this supplement was dissolved in 4 mL of sterile deionised water, enhancing the medium’s selectivity for *Trichomonas* by providing essential nutrients and growth factors.
- Sterile, inactivated horse serum: The serum, inactivated by heating at 56 °C for 30 min to eliminate any potential pathogens, was adjusted to a pH of 6 and added to the broth in a volume of 70 mL. This component is crucial for providing a rich source of additional nutrients and factors conducive to the protozoa’s growth.

These components were combined under sterile conditions to create an environment that closely mimics the protozoa’s natural habitat, thereby facilitating their survival and reproduction in a laboratory setting.

### Quantification of protozoa

The quantification of live trophozoites per millilitre was meticulously performed using a Burker chamber. This method employed a standardized formula commonly used for cell counting, ensuring accuracy and reproducibility in quantifying protozoal populations. The initial count was conducted following a 24-hour incubation period, after the samples arrived in the laboratory. Incubation then continued until the trophozoite population reached a sufficient concentration, specifically 10^6 cells/mL.

To calculate the trophozoite cell count, the formula applied was as follows: the average number of trophozoites observed across the 25 large squares of the Burker chamber was multiplied by the dilution factor, and then further multiplied by 2.5 × 10^5. This calculation can be represented as:

$$\:\frac{number\:of\:trophozoites\:in\:25\:large\:squares}{25}\:\times\:\:dilution\:factor\:\times\:\:2.5\:\times\:\:{10}^{5}$$

### Testing with proton pump inhibitors and nitroimidazoles

The efficacy of proton pump inhibitors - esomeprazole, lansoprazole, omeprazole, pantoprazole, and rabeprazole - against *Trichomonas gallinae* was investigated, utilizing stock solutions prepared from compounds supplied by Merck KGaA, Darmstadt, Germany. The solvents for these preparations varied; distilled water was used for pantoprazole and rabeprazole, while 96% ethanol (24% V/V) served as the solvent for esomeprazole, lansoprazole, and omeprazole, with all stock solutions standardized to a concentration of 8000 µg/mL. As a positive control, parasites’ susceptibility was assessed using four nitroimidazole compounds: metronidazole, ronidazole, tinidazole, and secnidazole (Merck KGaA, Darmstadt, Germany). To prepare the stock solutions at a concentration of 1024 µg/mL, we dissolved 30 mg of each active substance in 4.3 mL of dimethyl sulfoxide (DMSO) followed by dilution with 25 mL of distilled water, resulting in a final DMSO concentration of 14% (v/v) in the stock solutions. The in vitro data on nitroimidazole compounds were included solely for comparative reference purposes and did not constitute a primary objective of the study.

**Fig. 11:**
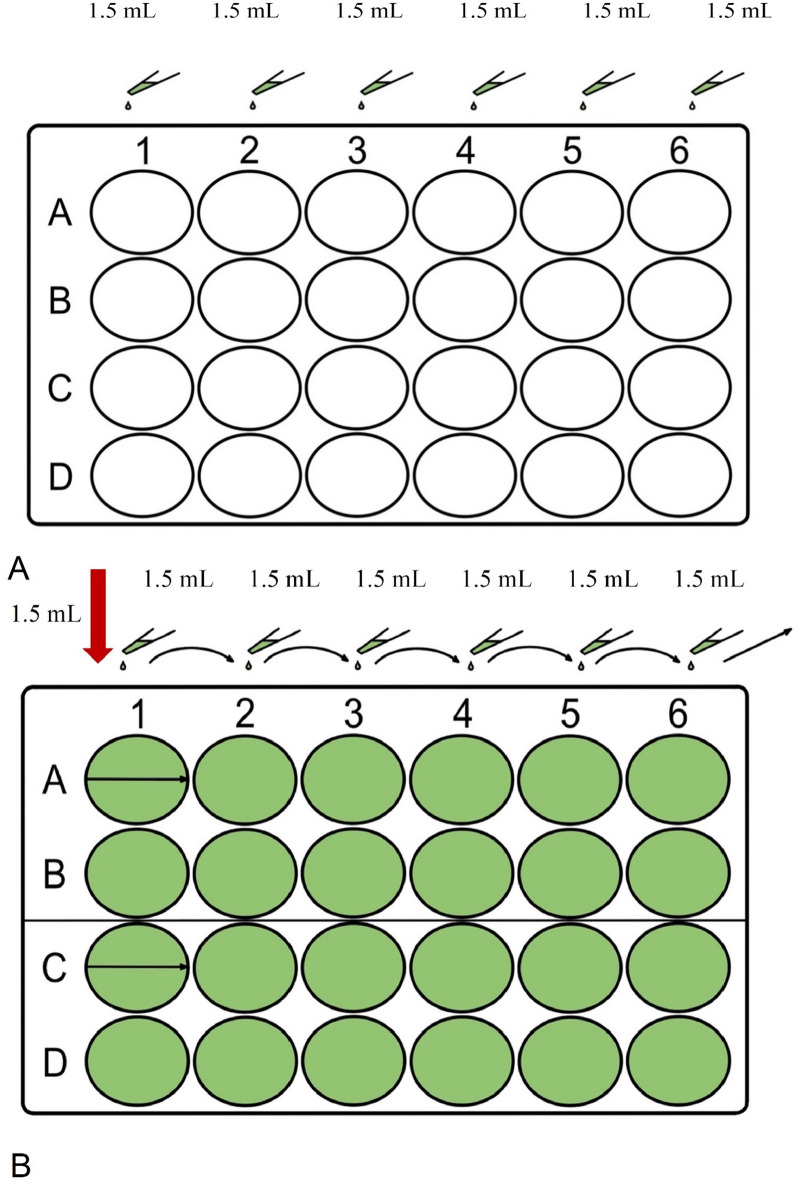
Preparation of 24-well plates for parasite culture and treatment. (**A**) In the first step, 1.5 mL of *Trichomonas* cysteine peptone liver infusion medium (CPLM) was added to each well of the 24-well plate. (**B**) In the second step, 1.5 mL of stock solution was added to the first well of the first row (A1) and the same volume of the corresponding solvent was added to the first well of the third row (C1). Serial two-fold dilutions of stock solution and solvent were prepared across wells A2 to B6 and C2 to D6, respectively.

Experimental treatments were conducted using 24-well cell culture plates (VWR International, LLC., Debrecen, Hungary), each initially filled with 1.5 mL of CPLM broth. To commence the concentration-response experiment, 1.5 mL of each proton-pump inhibitors and nitroimidazole stock solution was added to the first well in the first column of the plate, achieving a 2× dilution. This step was illustrated in Fig. 11A. From this starting point, a serial dilution (two-fold) was performed across the first two rows, as depicted in Fig. 11B, to establish a range of concentrations of the proton-pump inhibitors.

For proton-pump inhibitors dissolved in 96% ethanol, a parallel dilution series was prepared using the solvent alone. This control was designed to ascertain whether observed changes in trophozoite counts resulted from the effect of the proton-pump inhibitors or the ethanol. After completing the dilution series, excess fluid from the final dilution well was removed and discarded.

Subsequently, 100 µL of the *Trichomonas* suspension was added to each well and the plates were incubated at 37 °C, under a 5% CO_2_ atmosphere for 24 h. Following incubation, trophozoite numbers were assessed using a Burker chamber, with counts determined by averaging observations across five large squares.

In vitro susceptibility tests were performed on three separate occasions (*n* = 3), each conducted independently on different days using freshly prepared parasite cultures to ensure true biological replication. Within each experimental run, three technical replicates (triplicate wells per condition) were included and their values averaged to generate a single data point per concentration.

### Statistical analysis

Statistical analysis of data was conducted using R program version 4.1.0^46^. To assess the normality of the distribution, the Shapiro-Wilk test was employed. For datasets not adhering to a normal distribution, non-parametric tests were subsequently utilized. The Kruskal-Wallis test was leveraged to evaluate differences among the various compounds^47^. This test facilitates comparison across several sample groups by focusing on median values, rather than normal distribution, making it particularly suited for analyzing disparities between groups.

Further exploration of specific group correlations was achieved through post hoc analysis, employing the Mann-Whitney U test for pairwise comparisons^48^. Given the multiple comparisons made, we adjusted the resultant p-values using the Bonferroni correction to mitigate the risk of type I error inflation. It’s imperative to note, however, that the application of Bonferroni correction carries an inherent risk of augmenting second-order errors, namely the oversight of genuine differences.

Our statistical representation included box plots, detailing the mean, median, quartiles, and standard deviation for each active substance. Additionally, we calculated the average mortality rate attributable to each active substance and generated heat maps to visualize the percentage reduction in parasite numbers across varying concentrations of active substances. Lastly, we quantified the efficacy of each concentration in reducing parasite numbers for every active ingredient, providing a comprehensive overview of our findings.

The antiparasitic efficacy of the tested compounds was evaluated using a concentration-dependent inhibition assay. Each compound was tested in triplicate, and the number of surviving parasites was quantified following a 24-hour incubation period. The percentage of inhibition was calculated relative to the ethanol vehicle control (2,140,000 parasites) using the following formula: inhibition (%) = 100 × (1 – treated count / control count). Based on the resulting inhibition values, IC₅₀ estimates were obtained by fitting a four-parameter logistic (4PL) model^49^which is widely accepted for describing nonlinear, sigmoidal concentration–response relationships. Curve fitting was performed using nonlinear least squares regression via the *curve_fit* function from the SciPy Python package, with compound concentrations log-transformed prior to fitting. IC₅₀ values were derived by exponentiating the logIC₅₀ parameter obtained from the model. All data processing and curve visualizations were conducted using Python 3.10 with the *pandas*, *numpy*, *scipy*, and *matplotlib* libraries^50,51^.

## Conclusion

In conclusion, while nitroimidazole agents are primarily recognized and utilized for treating Trichomonas infections, which persist as significant concerns for both animal and public health. It is crucial to acknowledge that the use of these substances in pigeons is precluded, due to their designation as food-producing animals. However, the advance of benzimidazole-structured materials holds considerable promise for combating protozoan diseases. Among the evaluated proton pump inhibitors, omeprazole, pantoprazole, and esomeprazole have demonstrated superior effectiveness, with omeprazole standing out in particular for its efficacy. Proton pump inhibitors are already approved by the Food and Drug Administration (FDA), facilitating their in vivo testing in animals to assess efficacy. Given their current market availability and relative cost-effectiveness, proton pump inhibitors could serve as an efficacious alternative for treating *Trichomonas gallinae* infections in livestock, offering a viable solution to a pervasive health challenge.

## Electronic supplementary material

Below is the link to the electronic supplementary material.

Supplementary Material 1

## Data Availability

The datasets supporting the conclusions of this article are included within the article and its additional file.

## Abbreviations

CPLM: Cysteine peptone liver infusion medium
DMSO: Dimethyl sulfoxide
PPIs: Proton pump inhibitors
