# Supplementary material for: In vitro susceptibility testing of Trichomonas gallinae strains to proton pump inhibitors and nitroimidazoles
Source: Sci Rep. 2025 Jul 8;15:24437. doi: 10.1038/s41598-025-10668-w (PMC12238480; doi:10.1038/s41598-025-10668-w)
Supplement: Supplementary file 1 — Supplementary Material 1 [file 41598_2025_10668_MOESM1_ESM.zip › Supplementary materials.docx]

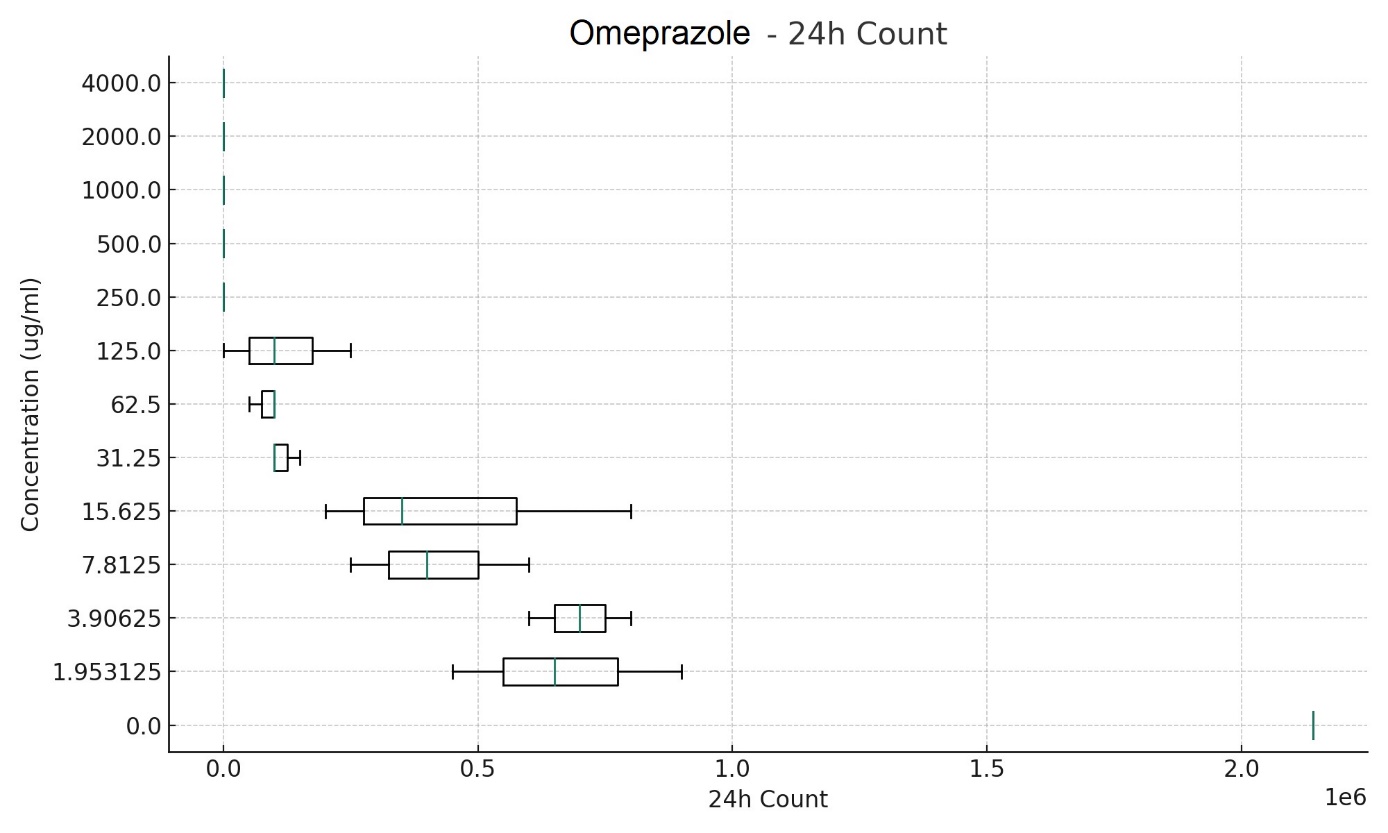


*Supplementary Figure 1* Concentration distribution of omeprazole during the 24-hour period of antiparasitic treatment. Boxplots show median concentration (horizontal line), interquartile range (boxes) and outliers (individual points above or below the whiskers). Data represents three independent experiments (*n*=3), each performed on a different day using separately cultured parasite populations. For each experimental run, measurements were taken from triplicate technical replicates and averaged. The figure helps to assess the variability of omeprazole doses administered during the treatment period.


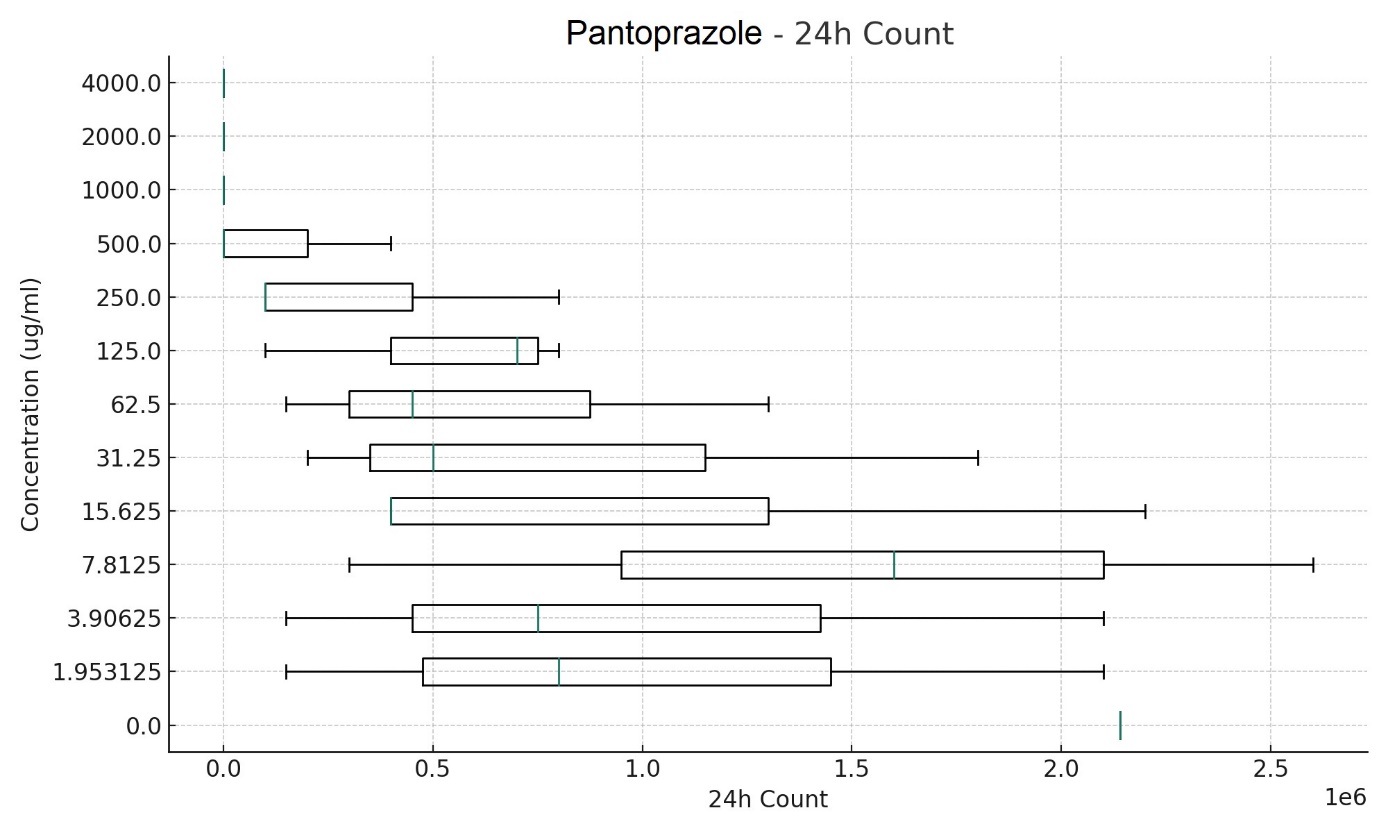


*Supplementary Figure 2* Pantoprazole concentration distribution over 24 hours. The boxplots show the median (horizontal line), interquartile range (boxes), and extreme values (whiskers), with outliers represented as individual points indicating unusually high or low concentrations. Data is based on three independent experiments (*n*=3), each conducted on separate days using freshly prepared parasite cultures. In each experiment, triplicate technical replicates were measured and averaged per condition.


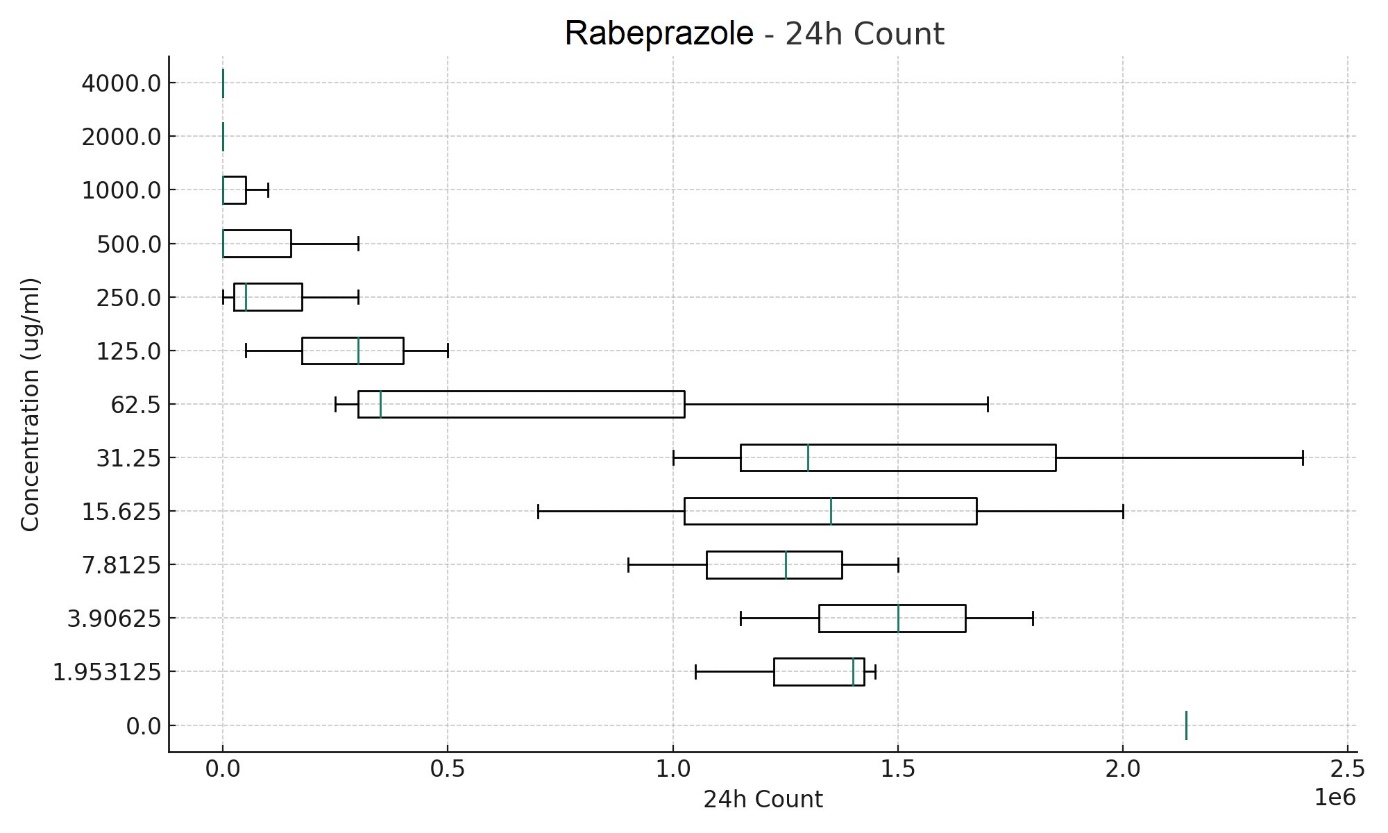


*Supplementary Figure 3* Rabeprazole concentration distribution over 24 hours. The boxplots show the median (horizontal line), interquartile range (boxes), and extreme values (whiskers), with outliers represented as individual points indicating extremely high or low concentrations. Data is derived from three independent experiments (*n*=3), each conducted on a separate day with independently cultured parasite samples. Each experiment included triplicate technical replicates per condition, which were averaged for analysis.


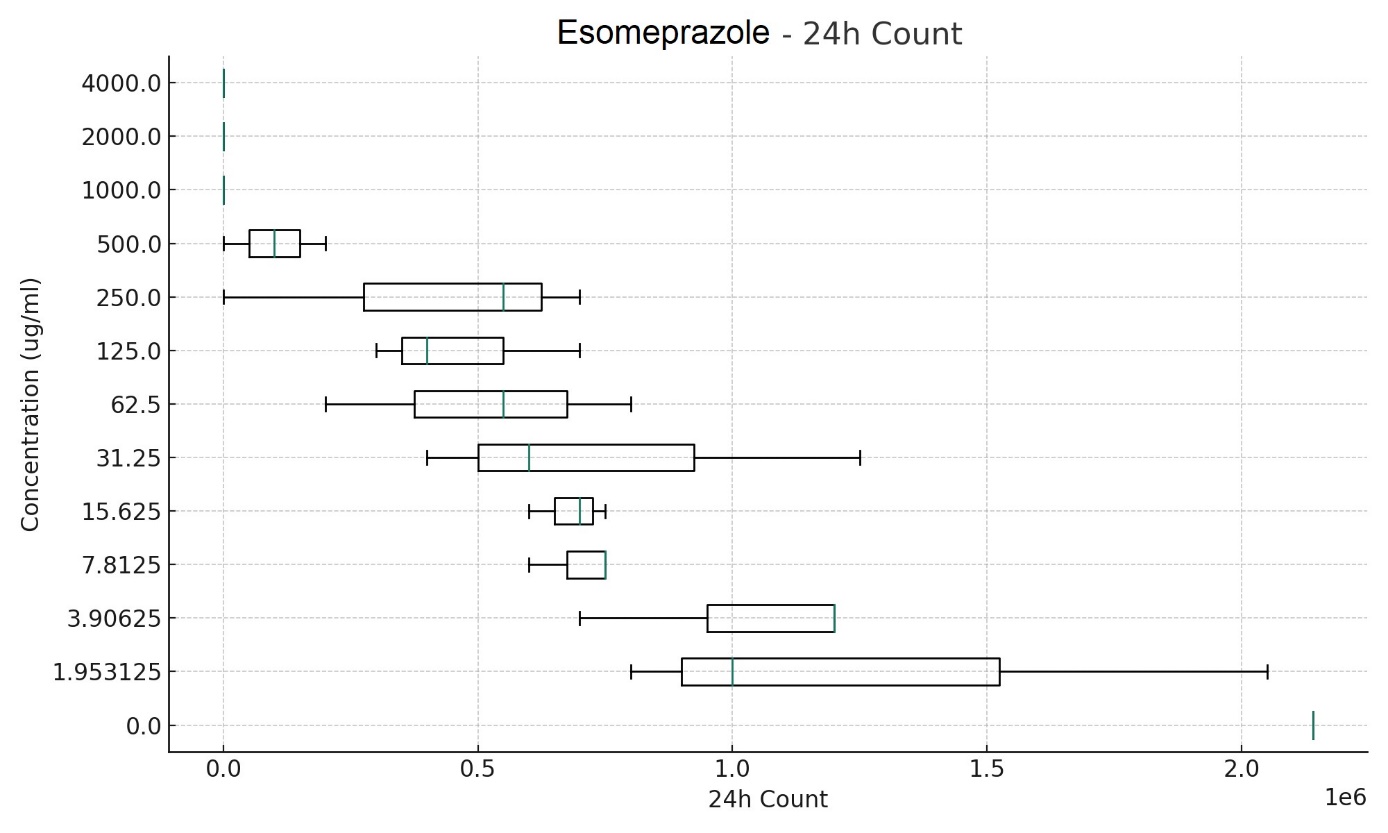


*Supplementary Figure 4* Esomeprazole concentration distribution over 24 hours. Boxplots show the median (horizontal line), interquartile range (boxes), and individual outliers representing particularly high or low concentrations. These visualizations reflect the central tendency and dispersion of the data. Relatively narrower boxplots indicate lower variability, while wider boxplots suggest higher variability among the data points. Data is based on three independent experiments (*n*=3), each conducted on different days with separately prepared parasite cultures. Triplicate technical replicates were included in each experiment and averaged for analysis.


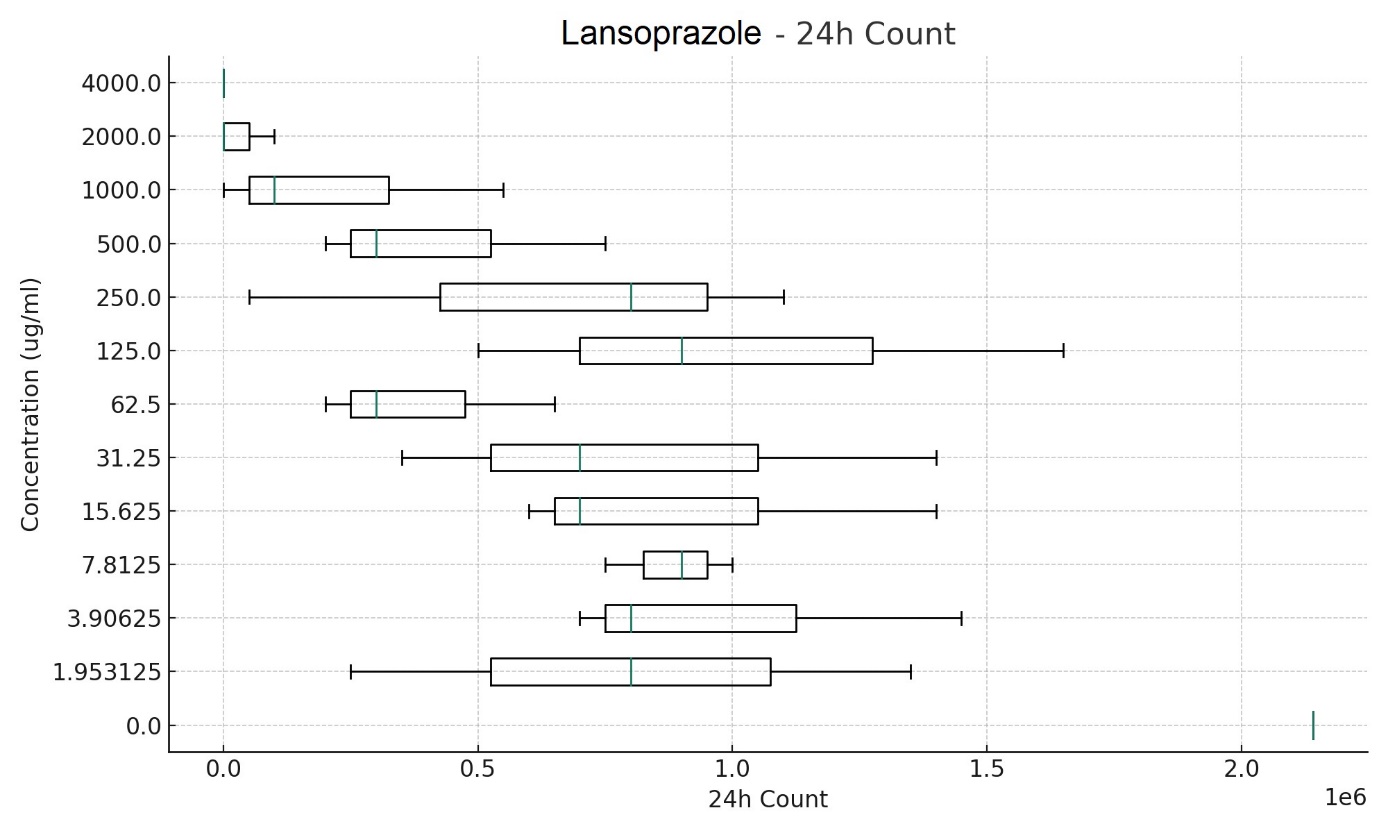


*Supplementary Figure 5* Concentration distribution of lansoprazole over 24 hours. Boxplots show the median (horizontal line), interquartile range (boxes), and total data range (whiskers), with outliers represented as individual points that deviate significantly from the overall distribution. Compared to previous cases, the variability is notably lower, indicating more consistent concentrations throughout the measurement period. Data are derived from three independent experiments (*n*=3), each performed on different days using distinct parasite cultures. Each experiment included triplicate technical replicates that were averaged per condition.


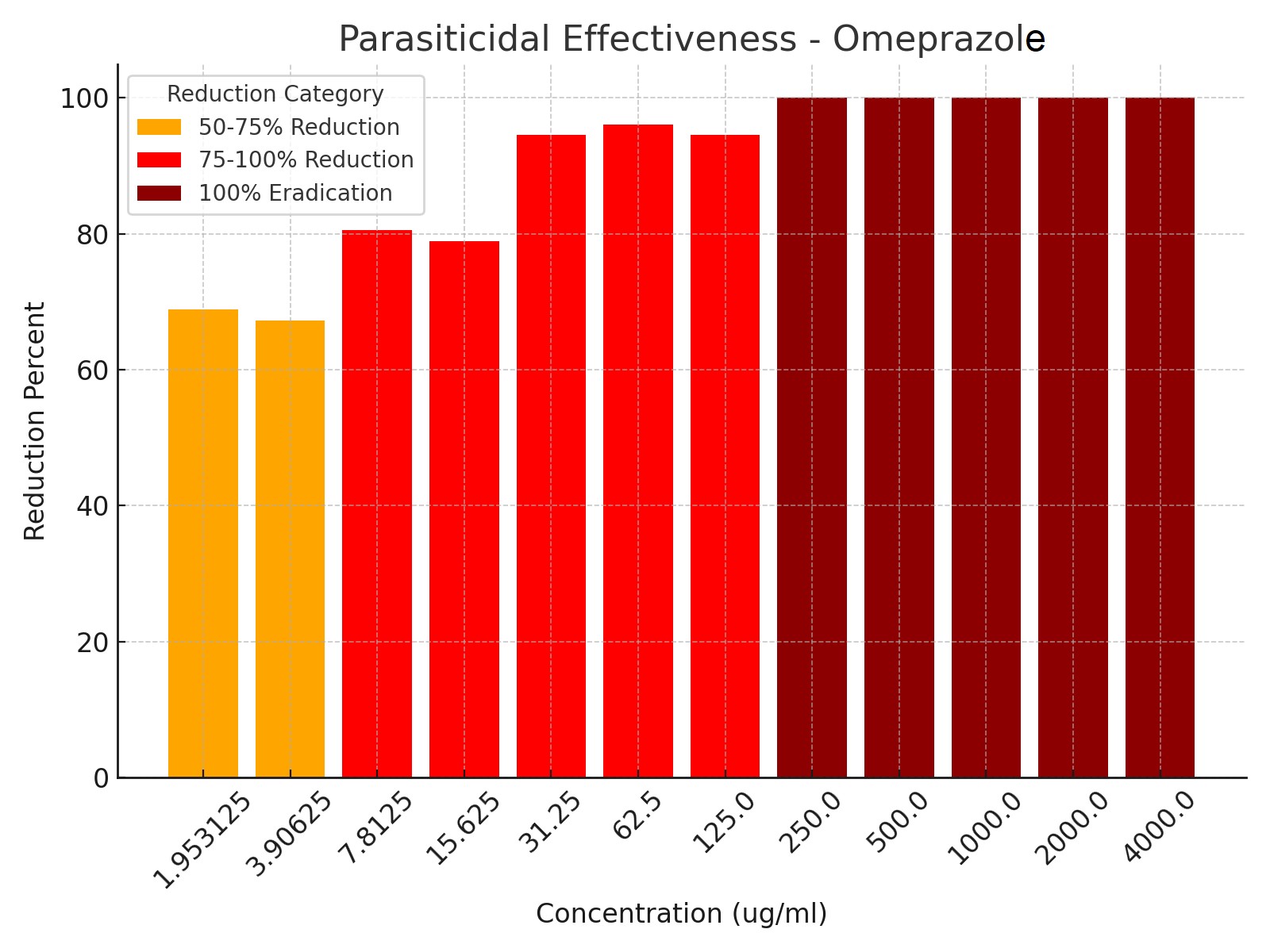


*Supplementary Figure 6* Antiparasitic efficacy of omeprazole at different concentrations. The graph shows the percentage reduction in parasite numbers following 24-hour incubation with increasing concentrations of omeprazole. Yellow bars indicate a reduction between 50–75%, orange bars represent 75–100% reduction, and red bars indicate complete (100%) eradication. Data represent means from three independent experiments (*n*=3), each conducted on a separate day with individually cultured parasite populations. Within each experiment, triplicate technical replicates were averaged for analysis. The graph demonstrates a clear concentration-dependent increase in antiparasitic efficacy.


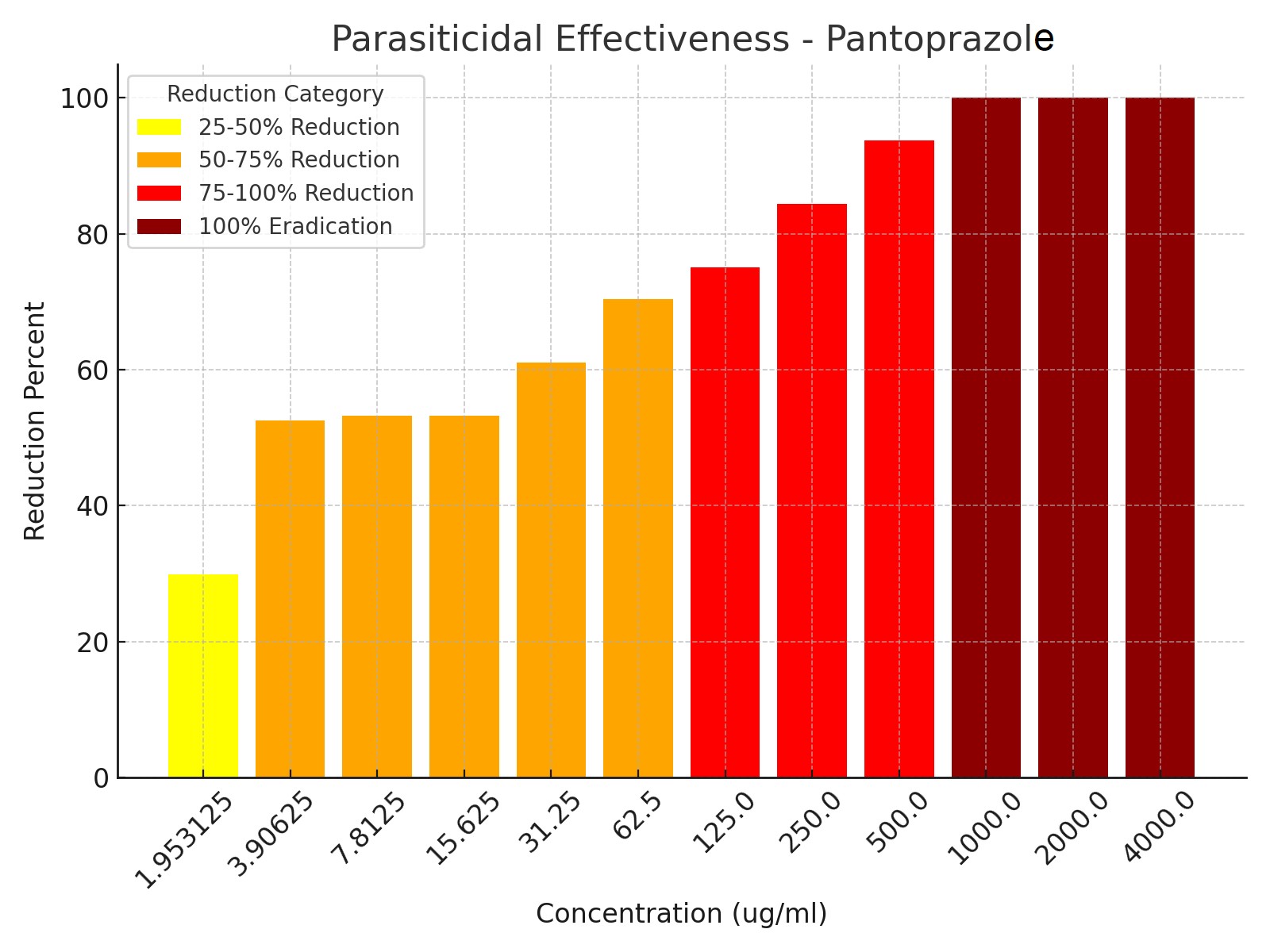


*Supplementary Figure 7* Antiparasitic efficacy of pantoprazole at different concentrations. The graph shows the percentage reduction in parasite numbers after 24-hour incubation with increasing concentrations of pantoprazole. Light-yellow bars represent a reduction of 25–50%, yellow bars represent 50–75%, orange bars indicate 75–100%, and red bars indicate complete (100%) eradication.
Data reflect the means of three independent experiments (*n*=3), each performed on separate days using distinct parasite cultures. Triplicate technical replicates were included and averaged for each experiment. The results demonstrate a clear concentration-dependent increase in antiparasitic efficacy at higher concentrations.


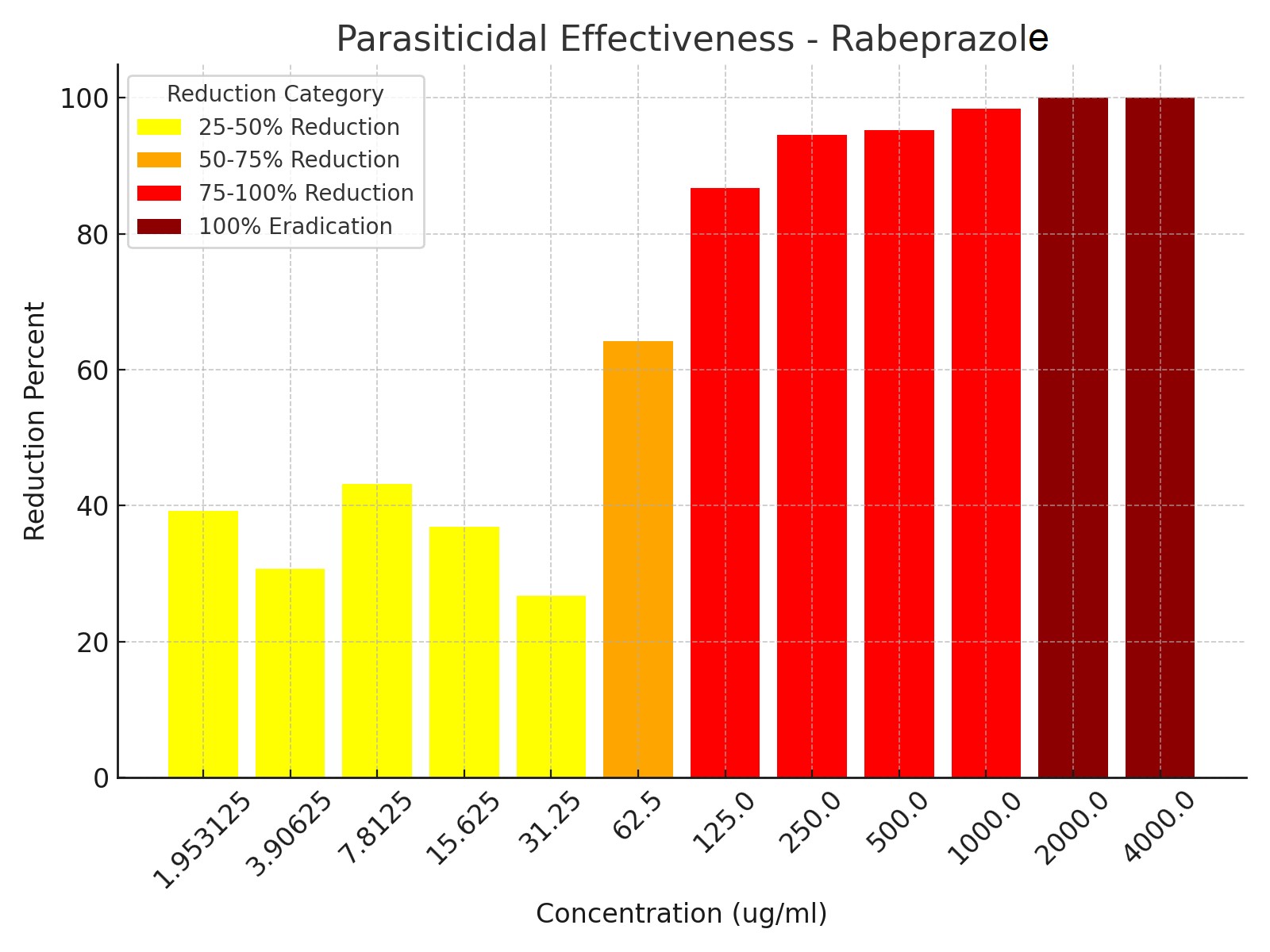


*Supplementary Figure 8* Antiparasitic efficacy of rabeprazole at different concentrations. The figure shows the percentage reduction in parasite numbers following 24-hour incubation with increasing concentrations of rabeprazole. Light-yellow bars indicate a 25–50% reduction, yellow bars 50–75%, orange bars 75–100%, and red bars represent complete (100%) eradication. Data represents the means of three independent experiments (*n*=3), each conducted on different days using freshly cultured parasites. Triplicate technical replicates were averaged within each experiment. The results demonstrate that the antiparasitic efficacy of rabeprazole increases markedly at higher concentrations.


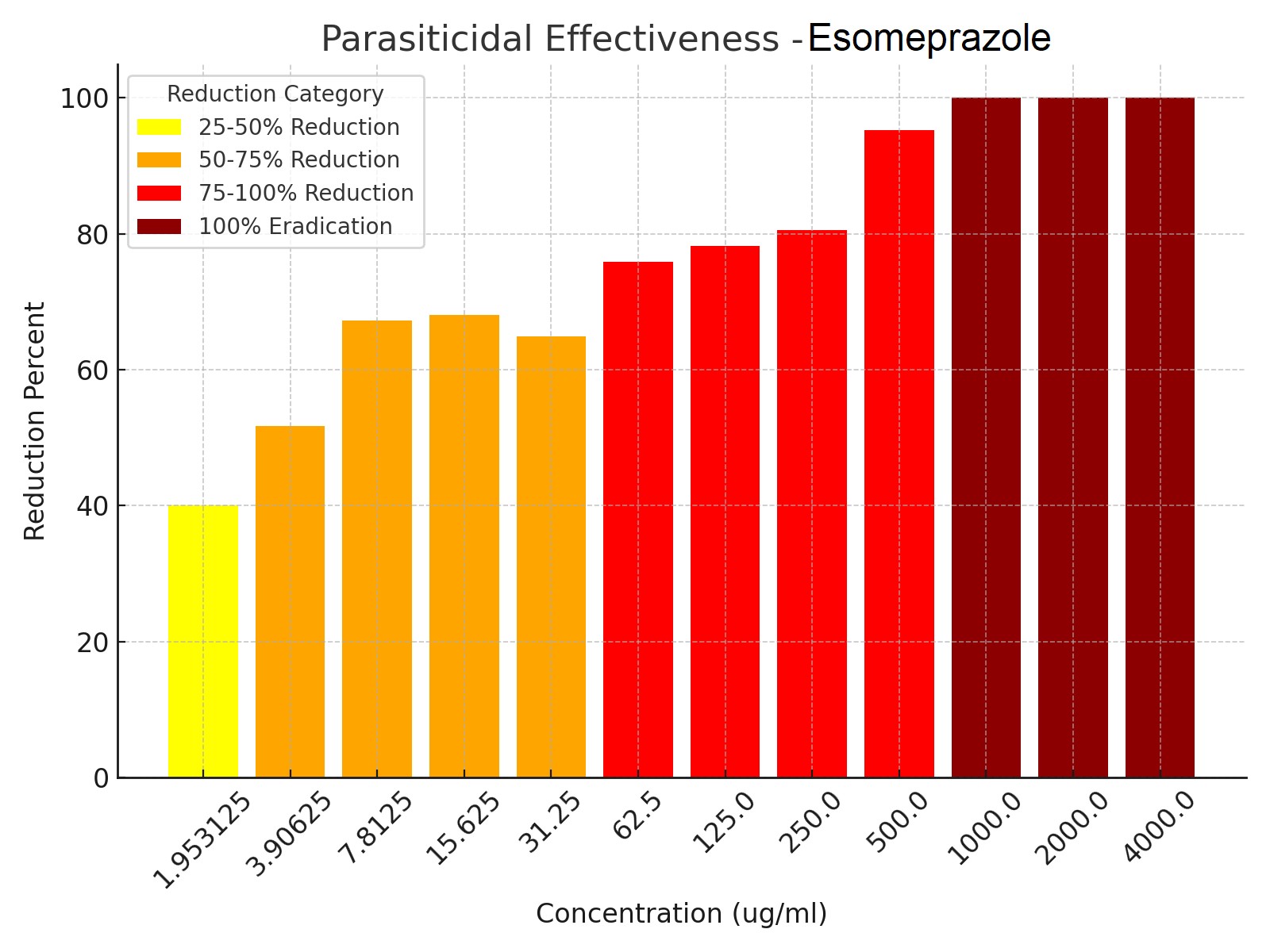


*Supplementary Figure 9* Antiparasitic efficacy of esomeprazole at different concentrations. The graph shows a concentration-dependent decrease in the number of parasites after 24-hour incubation with esomeprazole. Light-yellow bars indicate a 25–50% reduction, yellow bars 50–75%, orange bars 75–100%, and red bars indicate complete (100%) eradication. Data is presented as a means from three independent experiments (*n*=3), each conducted on different days using distinct parasite cultures. In each experiment, triplicate technical replicates were averaged per concentration.


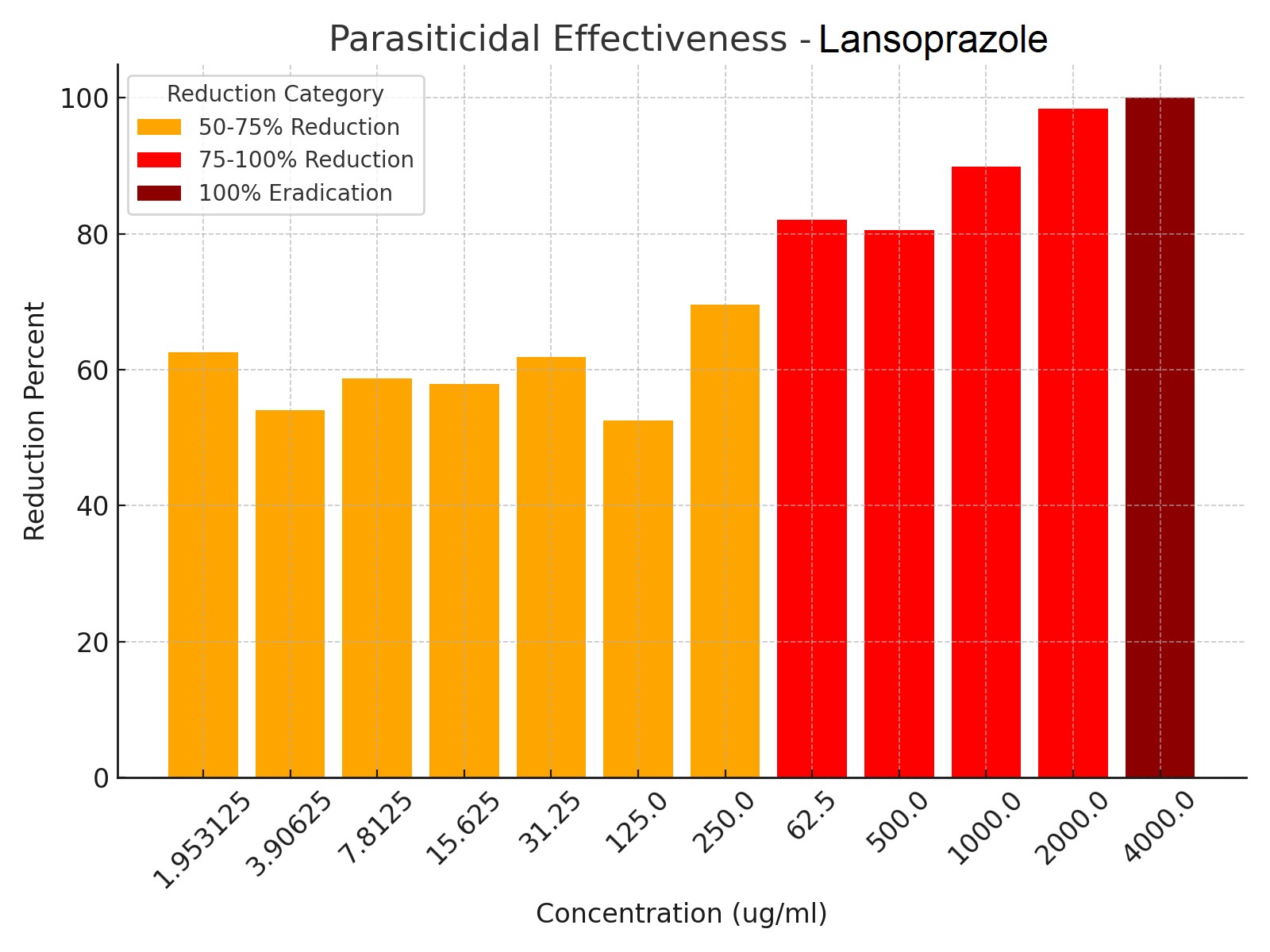


*Supplementary Figure 10* Antiparasitic efficacy of lansoprazole at different concentrations. The graph illustrates a concentration-dependent decrease in parasite numbers following 24-hour incubation with lansoprazole. Yellow bars represent a 50–75% reduction, orange bars indicate 75–100% reduction, and red bars signify complete (100%) eradication. Results are based on three independent experiments (*n*=3), each conducted on different days using separately cultured parasite samples. Triplicate technical replicates were included in each run and averaged for each concentration.


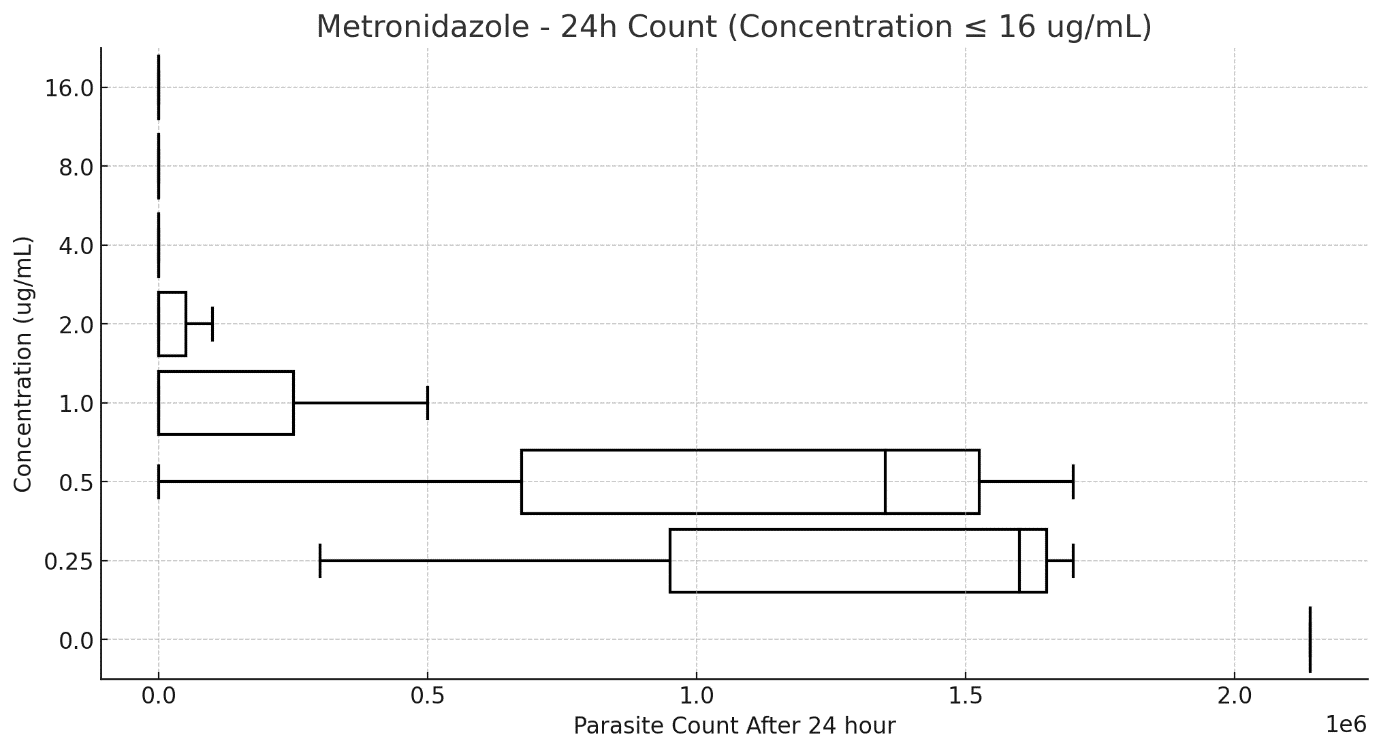


*Supplementary Figure 11* Effect of varied concentrations of metronidazole on the reduction of parasite numbers after 24 hours, illustrated using boxplots. Boxplots show the distribution of parasite reduction across different concentrations of metronidazole, based on 24-hour incubation.
The plots display median values, interquartile ranges (boxes), and outliers (individual data points beyond whiskers), highlighting variability in antiparasitic response. Data are derived from three independent experiments (*n* = 3), each conducted on a separate day using independently cultured parasite populations. Triplicate technical replicates were averaged within each experiment.


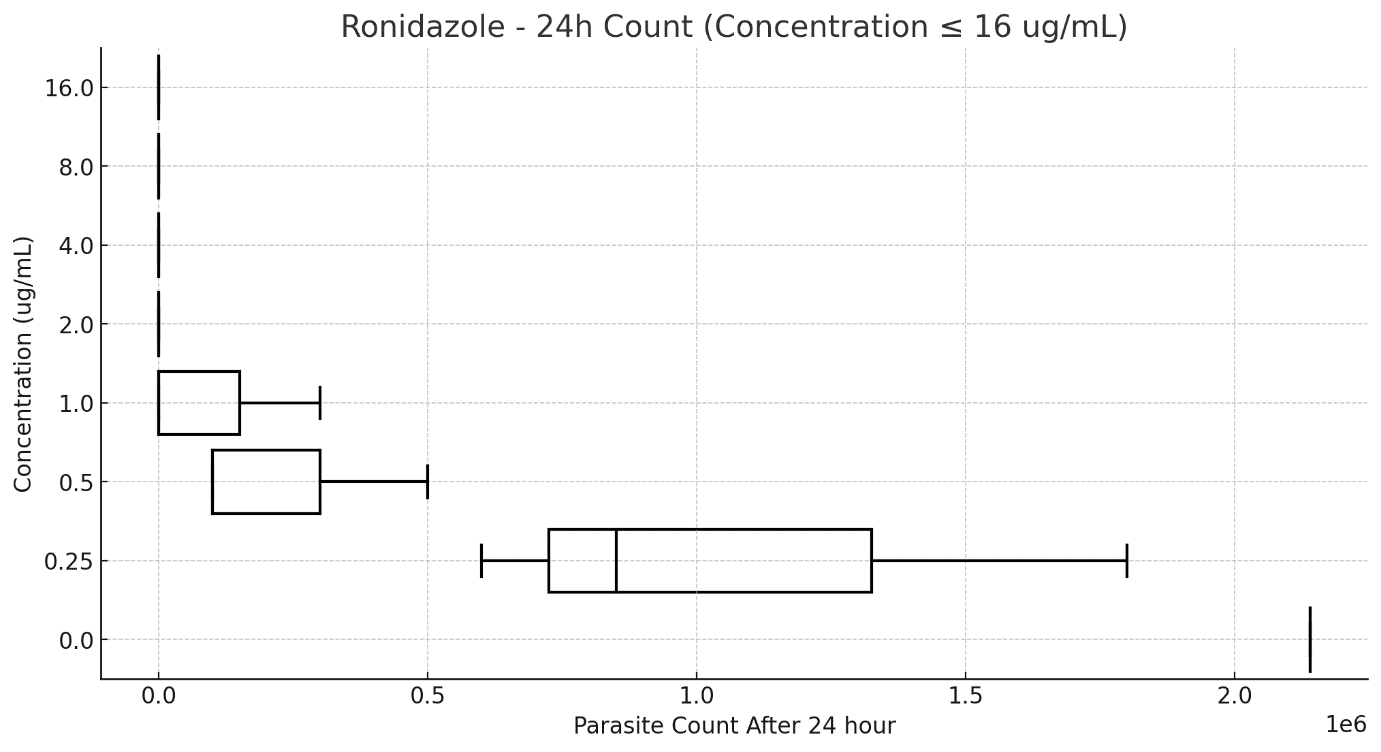


*Supplementary Figure 12* Effect of varied concentrations of ronidazole on the reduction of parasite numbers after 24 hours, illustrated using boxplots. Boxplots show the distribution of parasite reduction across different concentrations of metronidazole, based on 24-hour incubation.
The plots display median values, interquartile ranges (boxes), and outliers (individual data points beyond whiskers), highlighting variability in antiparasitic response. Data are derived from three independent experiments (*n* = 3), each conducted on a separate day using independently cultured parasite populations. Triplicate technical replicates were averaged within each experiment.


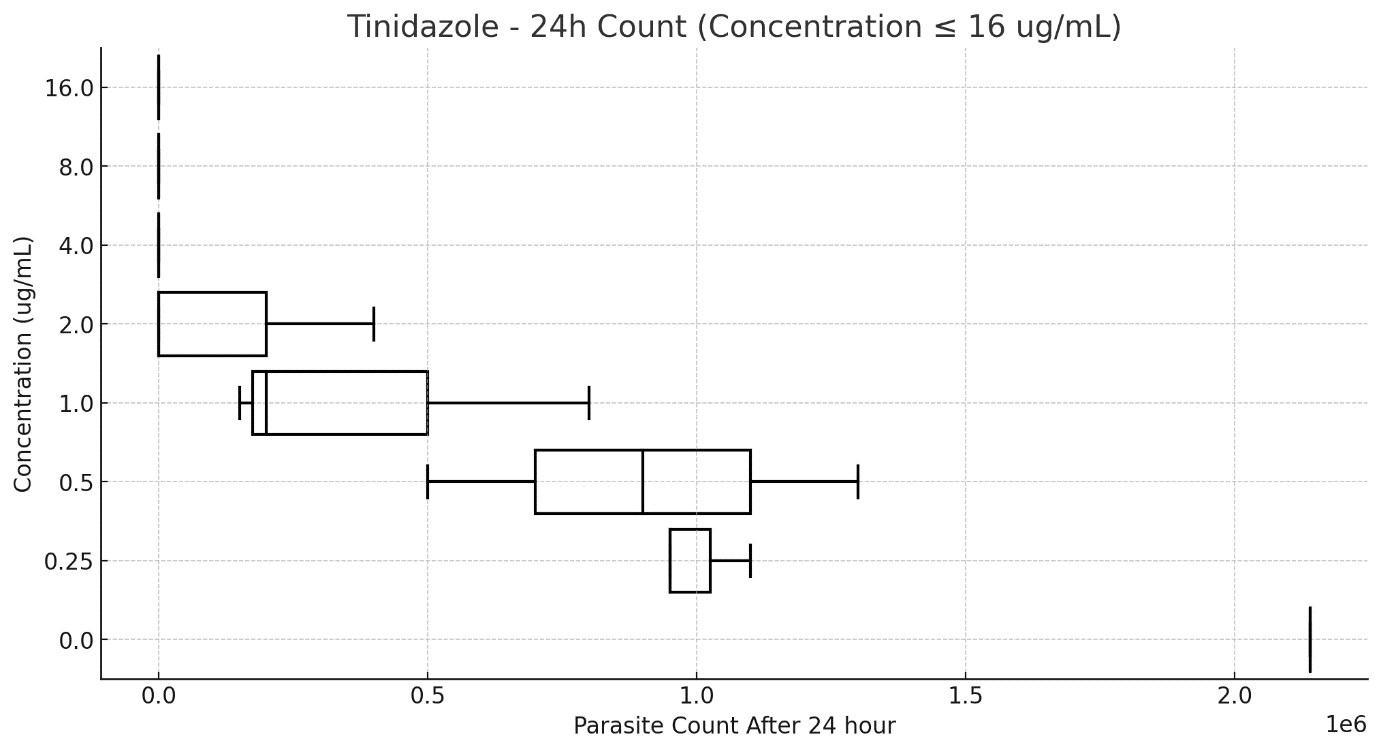


*Supplementary Figure 13* Effect of varied concentrations of tinidazole on the reduction of parasite numbers after 24 hours, illustrated using boxplots. Boxplots show the distribution of parasite reduction across different concentrations of metronidazole, based on 24-hour incubation.
The plots display median values, interquartile ranges (boxes), and outliers (individual data points beyond whiskers), highlighting variability in antiparasitic response. Data are derived from three independent experiments (*n* = 3), each conducted on a separate day using independently cultured parasite populations. Triplicate technical replicates were averaged within each experiment.


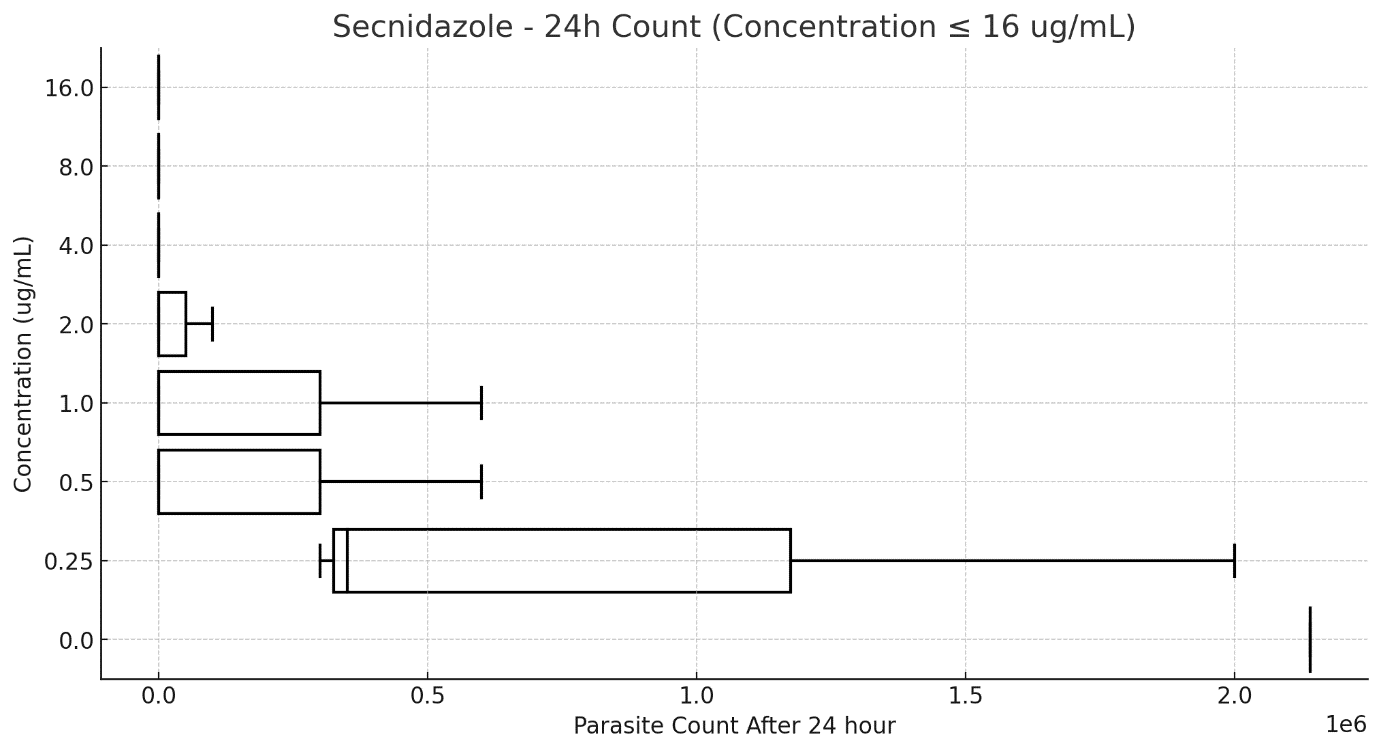


*Supplementary Figure 14* Effect of varied concentrations of secnidazole on the reduction of parasite numbers after 24 hours, illustrated using boxplots. Boxplots show the distribution of parasite reduction across different concentrations of metronidazole, based on 24-hour incubation.
The plots display median values, interquartile ranges (boxes), and outliers (individual data points beyond whiskers), highlighting variability in antiparasitic response. Data are derived from three independent experiments (*n* = 3), each conducted on a separate day using independently cultured parasite populations. Triplicate technical replicates were averaged within each experiment.

*Supplementary Table 1* Exact p-values for statistical comparisons between each concentration of the tested proton pump inhibitors and the untreated control group. Statistical significance was assessed using unpaired two-tailed t-tests. 'n.d.' indicates cases where statistical testing was not performed due to zero variance in the treated group (all replicates = 0).

| **Compound** | **Concentration (µg/mL)** | | | | | | | | | | | |
| --- | --- | --- | --- | --- | --- | --- | --- | --- | --- | --- | --- | --- |
|  | **1.95** | **3.91** | **7.81** | **15.63** | **31.25** | **62.5** | **125** | **250** | **500** | **1000** | **2000** | **4000** |
|  | **p-values** | | | | | | | | | | | |
| Omeprazole | 0.0077 | 0.0016 | 0.0034 | 0.0112 | <0.0001 | <0.0001 | 0.0013 | n.d. | n.d. | n.d. | n.d. | n.d. |
| Pantoprazole | 0.1891 | 0.1867 | 0.4379 | 0.1978 | 0.1170 | 0.0485 | 0.0180 | 0.0163 | 0.0044 | n.d. | n.d. | n.d. |
| Rabeprazole | 0.0217 | 0.0730 | 0.0337 | 0.1699 | 0.3103 | 0.0990 | 0.0049 | 0.0021 | 0.0024 | 0.0003 | n.d. | n.d. |
| Esomeprazole | 0.1577 | 0.0219 | 0.0012 | 0.0009 | 0.0324 | 0.0113 | 0.0051 | 0.0149 | 0.0008 | n.d. | n.d. | n.d. |
| Lansoprazole | 0.0518 | 0.0389 | 0.0033 | 0.0388 | 0.0503 | 0.0060 | 0.0795 | 0.0412 | 0.0095 | 0.0076 | 0.0003 | n.d. |

*Supplementary Table 2* Exact p-values for pairwise comparisons of nitroimidazole-treated samples versus the untreated control at each tested concentration. Analyses were based on three biological replicates per group. Statistical tests were performed using unpaired two-tailed t-tests where applicable. 'n.d.' indicates comparisons not applicable due to uniform zero values in the treated samples.

| **Compound** | **Concentration (µg/mL)** | | | | | | | | | | | |
| --- | --- | --- | --- | --- | --- | --- | --- | --- | --- | --- | --- | --- |
|  | **0.25** | **0.5** | **1** | **2** | **4** | **8** | **16** | **32** | **64** | **128** | **256** | **512** |
|  | **p-values** | | | | | | | | | | | |
| Metronidazole | 0.1725 | 0.1625 | 0.0071 | 0.0003 | n.d. | n.d. | n.d. | n.d. | n.d. | n.d. | n.d. | n.d. |
| Ronidazole | 0.1017 | 0.0049 | 0.0024 | n.d. | n.d. | n.d. | n.d. | n.d. | n.d. | n.d. | n.d. | n.d. |
| Tinidazole | 0.0019 | 0.0330 | 0.0138 | 0.0044 | n.d. | n.d. | n.d. | n.d. | n.d. | n.d. | n.d. | n.d. |
| Secnidazole | 0.1534 | 0.0105 | 0.0105 | 0.0003 | n.d. | n.d. | n.d. | n.d. | n.d. | n.d. | n.d. | n.d. |
