# Supplementary figures and images for: In vitro susceptibility testing of Trichomonas gallinae strains to proton pump inhibitors and nitroimidazoles
Source: Sci Rep. 2025 Jul 8;15:24437. doi: 10.1038/s41598-025-10668-w (PMC12238480; doi:10.1038/s41598-025-10668-w)

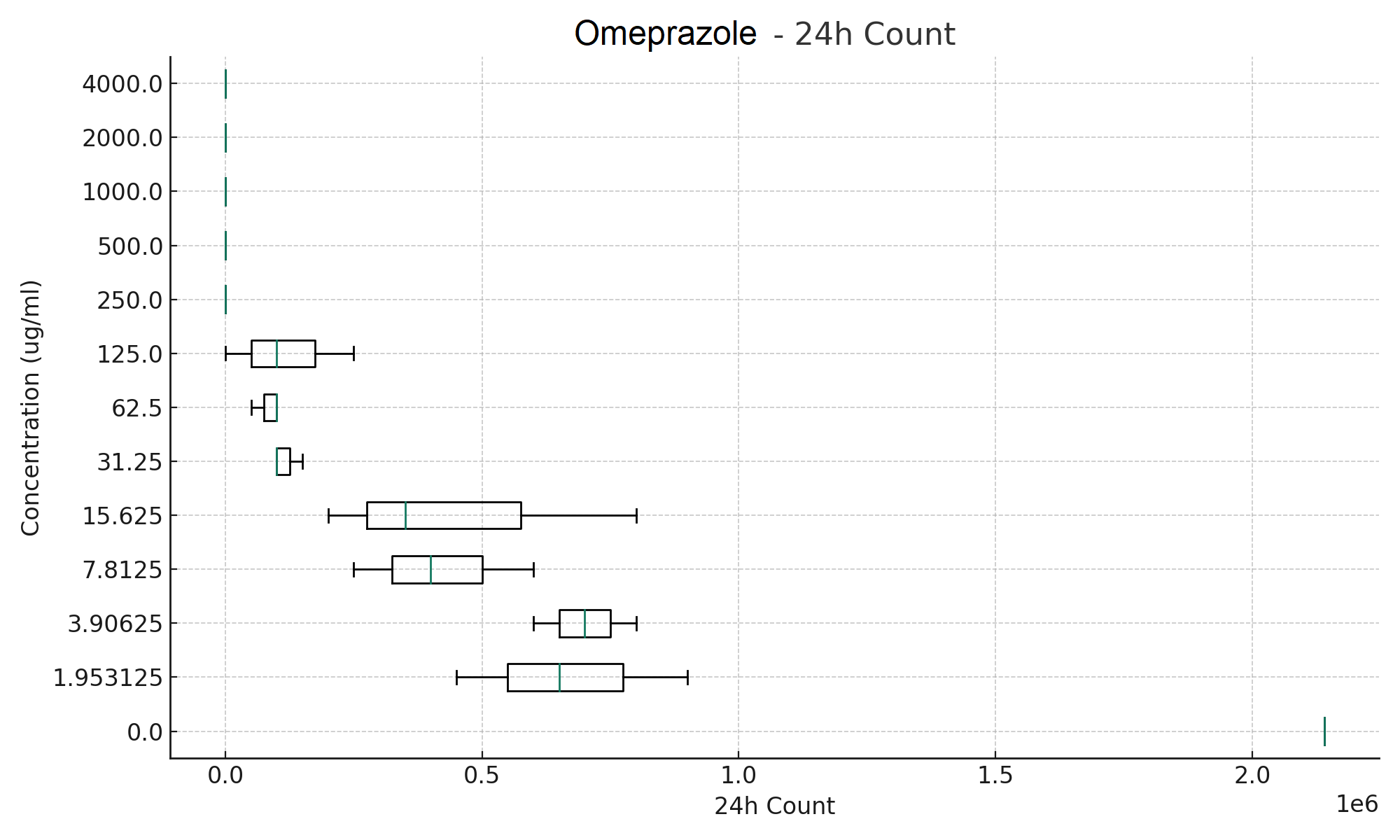

Supplement: Supplementary file 1 — Supplementary Material 1 [file 41598_2025_10668_MOESM1_ESM.zip › Supplementary Figure S1.png]

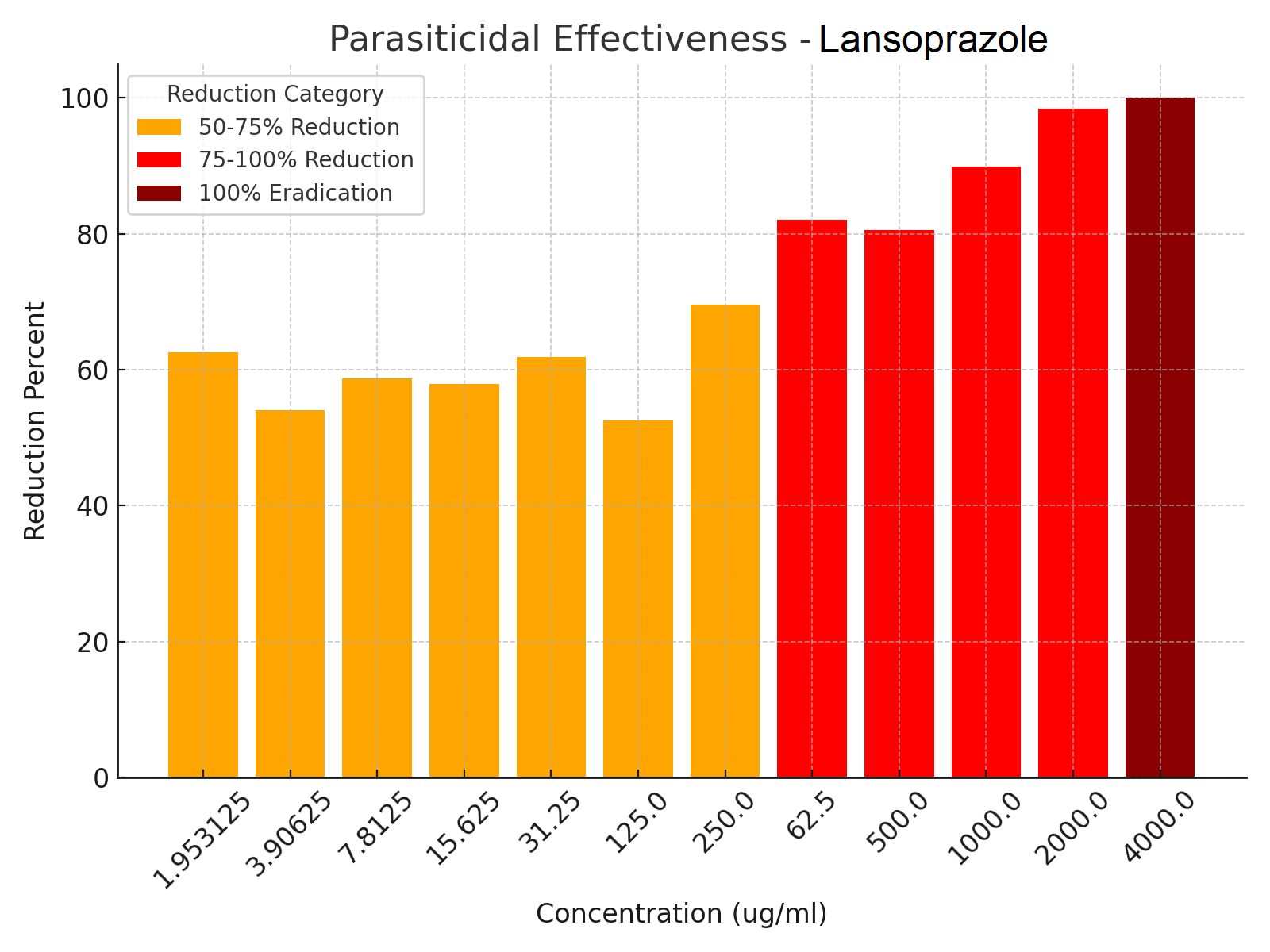

Supplement: Supplementary file 1 — Supplementary Material 1 [file 41598_2025_10668_MOESM1_ESM.zip › Supplementary Figure S10.png]

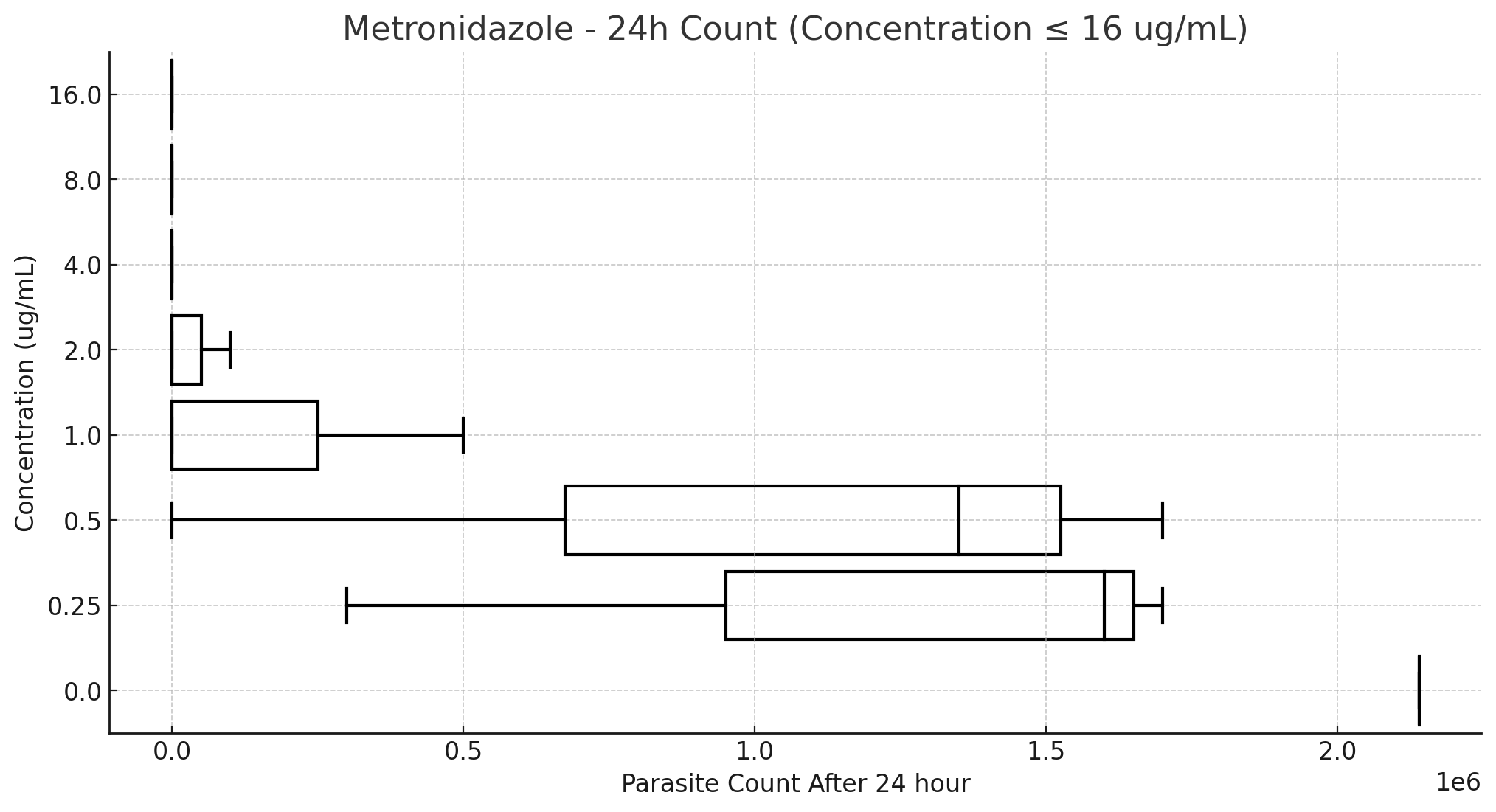

Supplement: Supplementary file 1 — Supplementary Material 1 [file 41598_2025_10668_MOESM1_ESM.zip › Supplementary Figure S11.png]

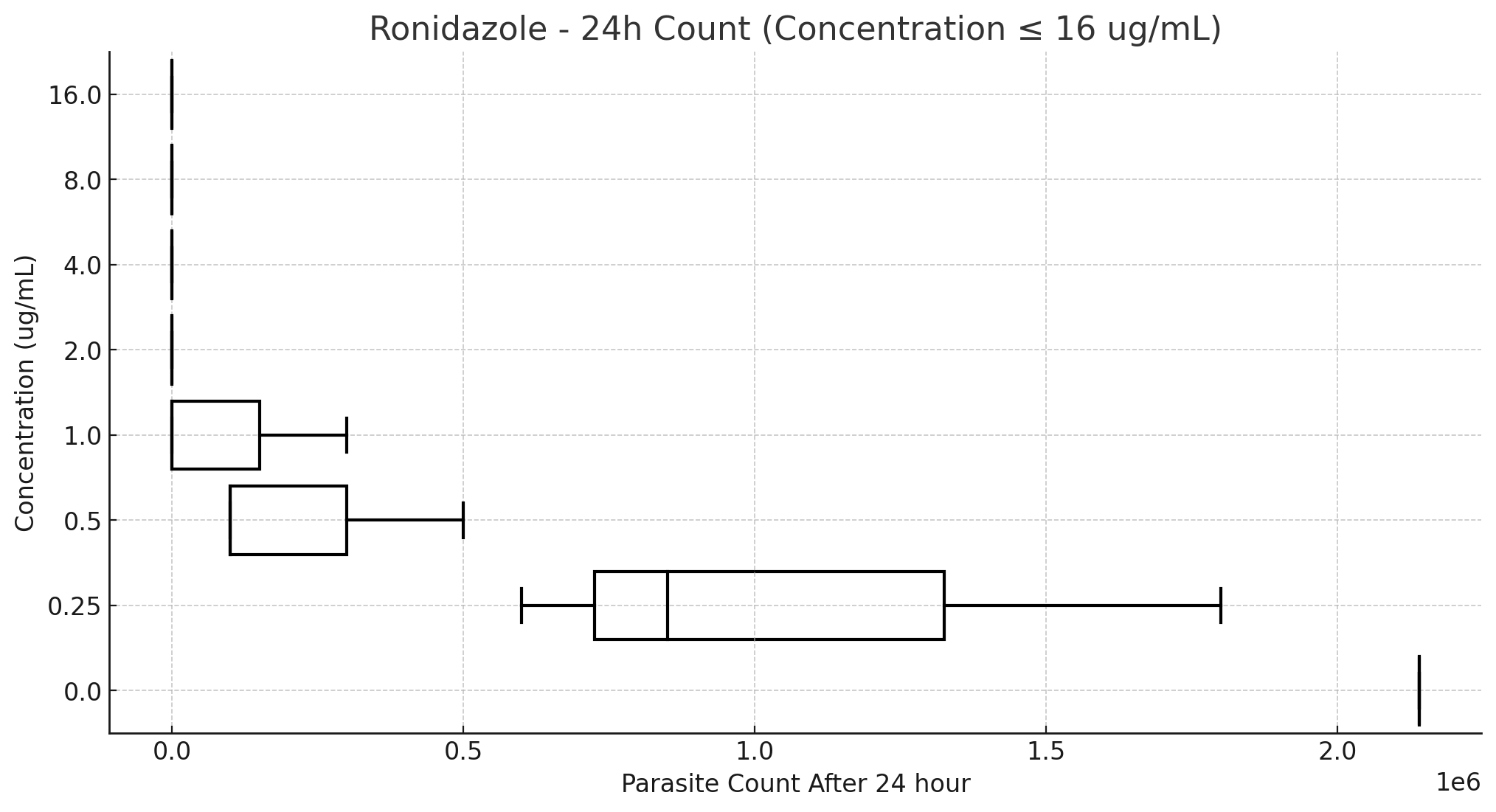

Supplement: Supplementary file 1 — Supplementary Material 1 [file 41598_2025_10668_MOESM1_ESM.zip › Supplementary Figure S12.png]

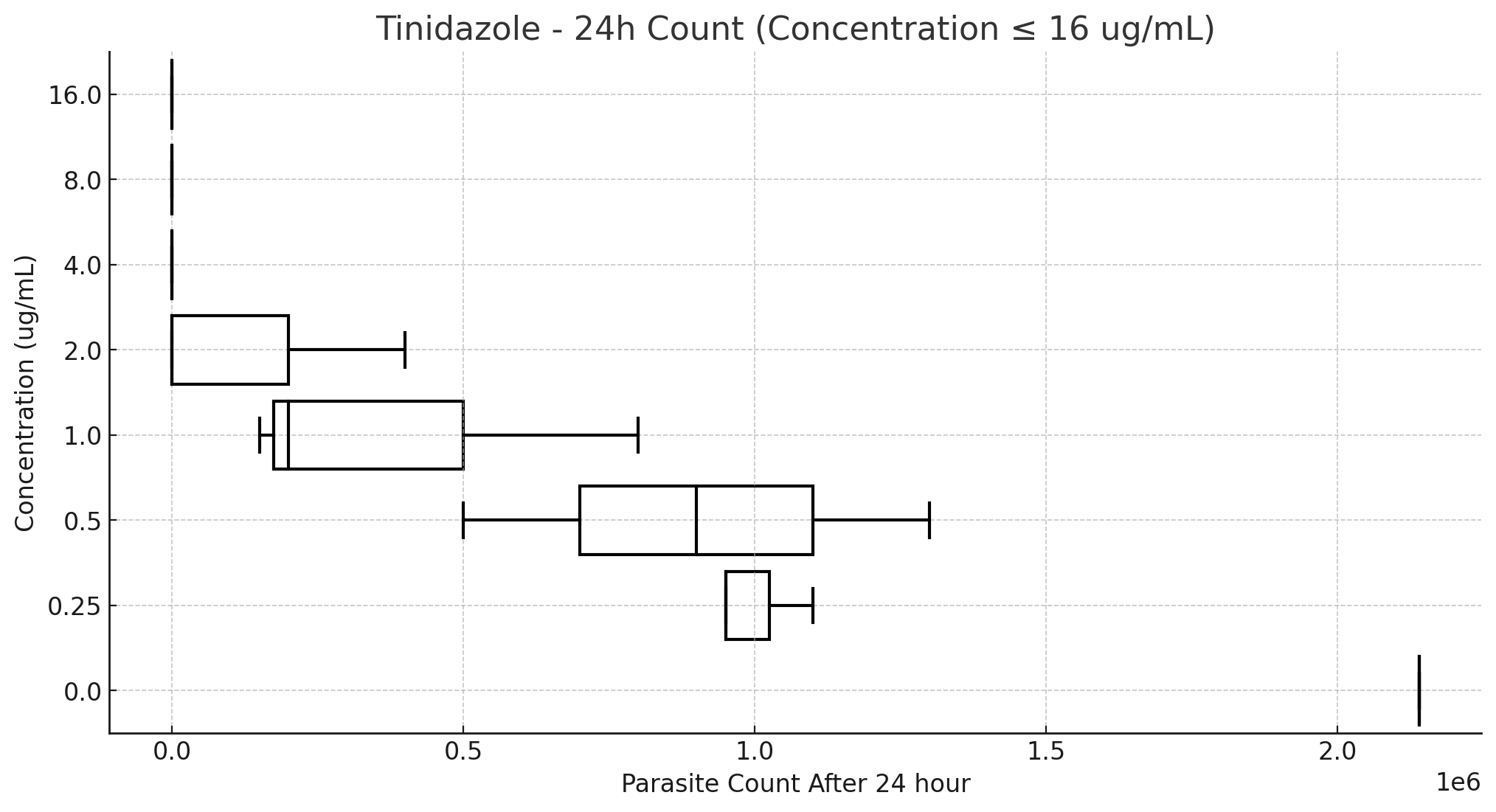

Supplement: Supplementary file 1 — Supplementary Material 1 [file 41598_2025_10668_MOESM1_ESM.zip › Supplementary Figure S13.png]

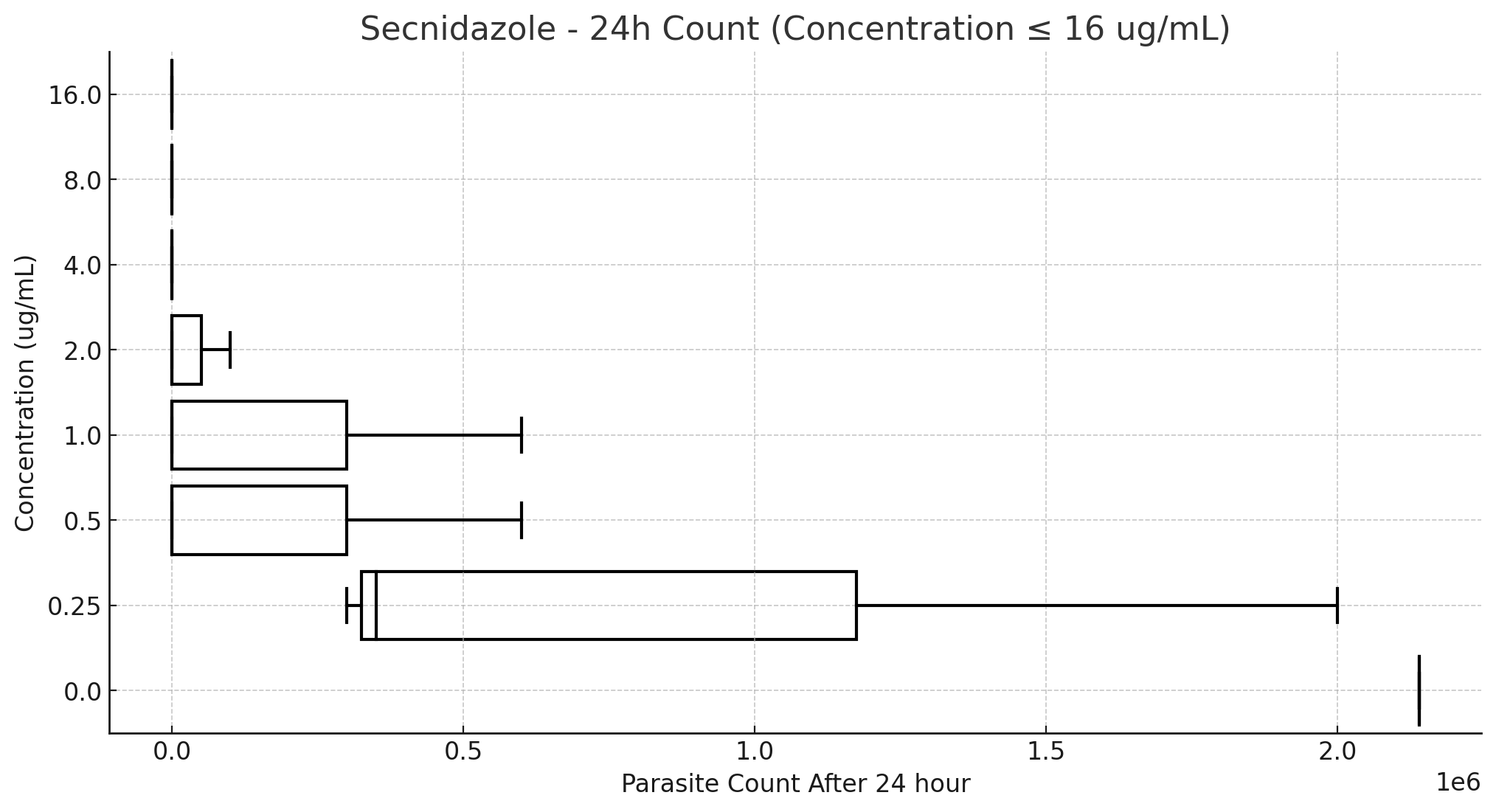

Supplement: Supplementary file 1 — Supplementary Material 1 [file 41598_2025_10668_MOESM1_ESM.zip › Supplementary Figure S14.png]

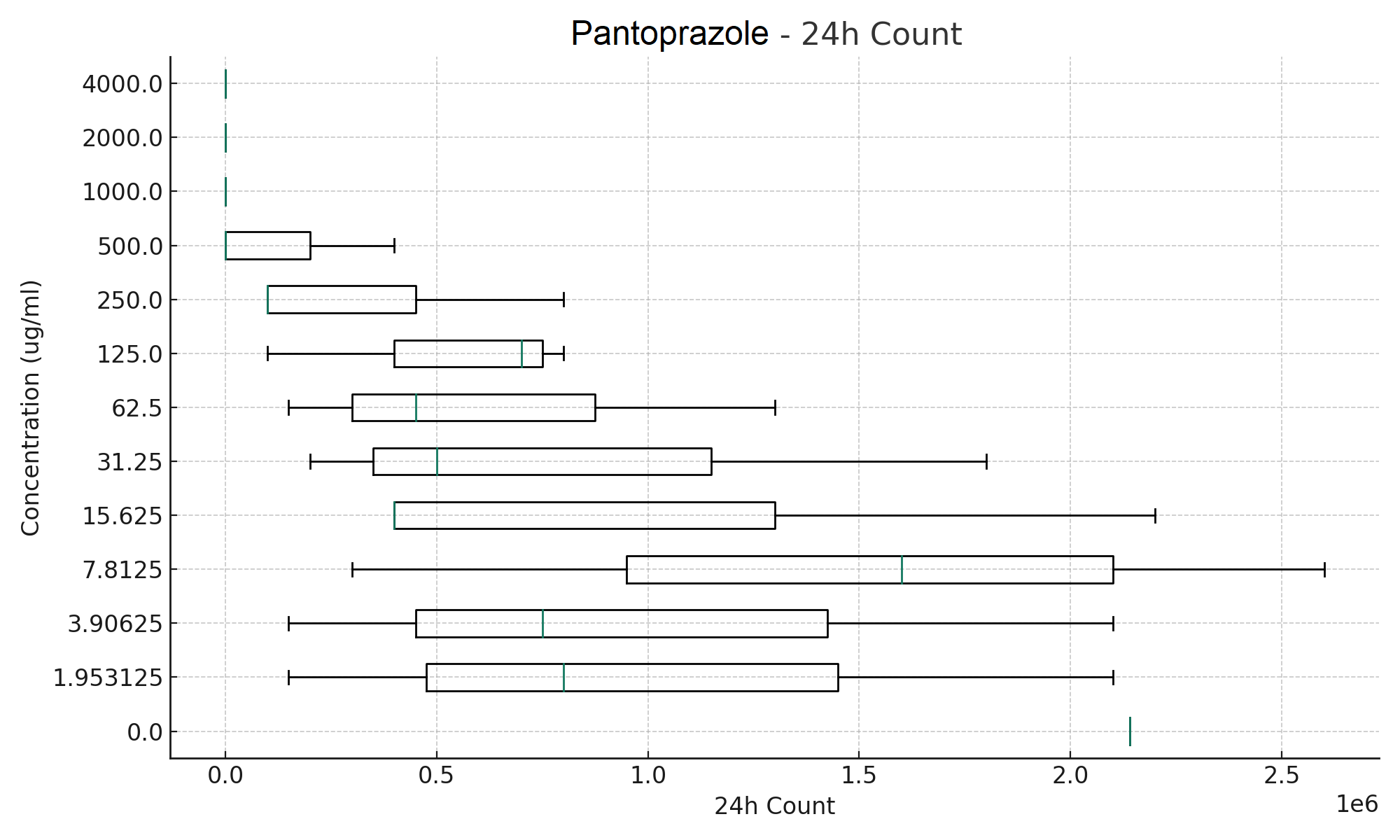

Supplement: Supplementary file 1 — Supplementary Material 1 [file 41598_2025_10668_MOESM1_ESM.zip › Supplementary Figure S2.png]

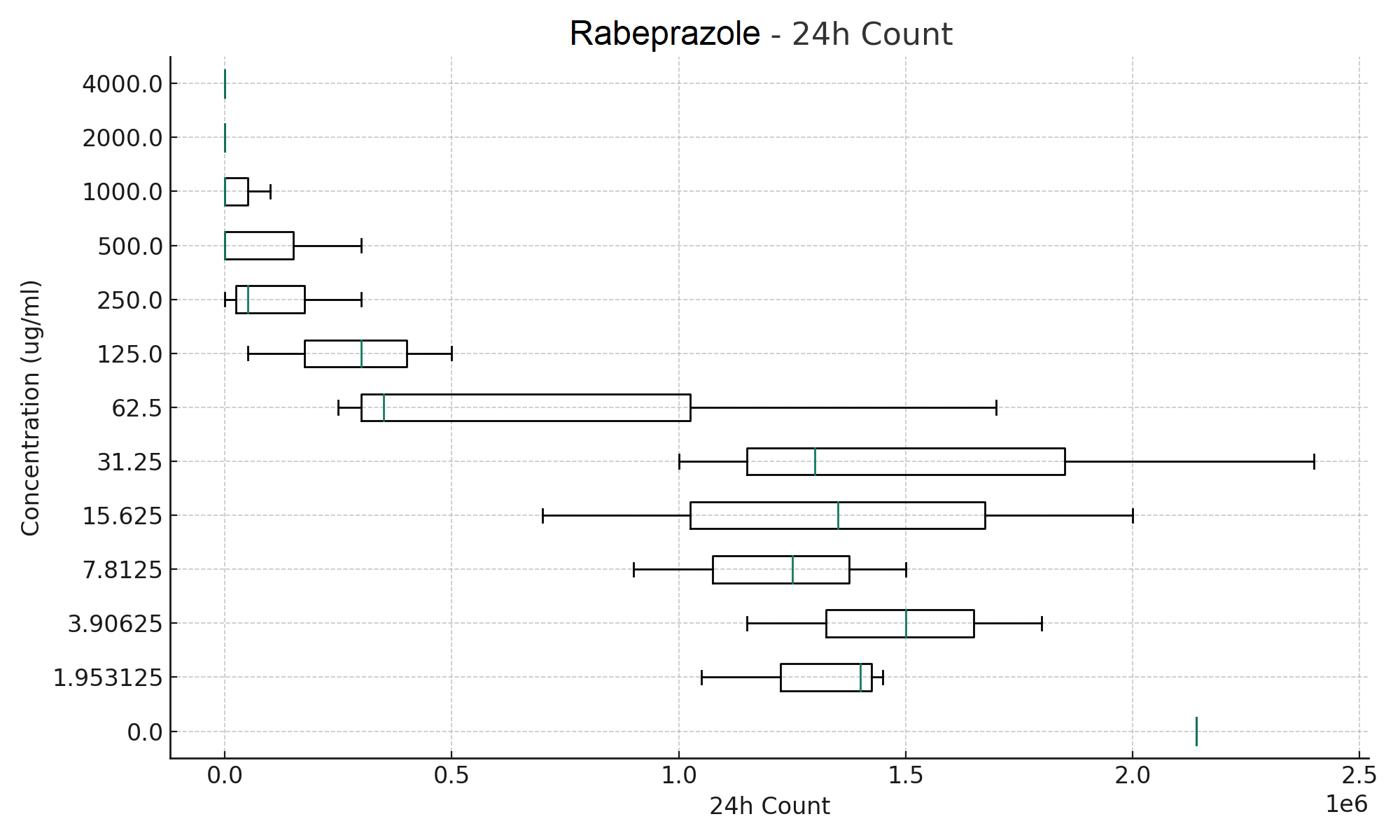

Supplement: Supplementary file 1 — Supplementary Material 1 [file 41598_2025_10668_MOESM1_ESM.zip › Supplementary Figure S3.png]

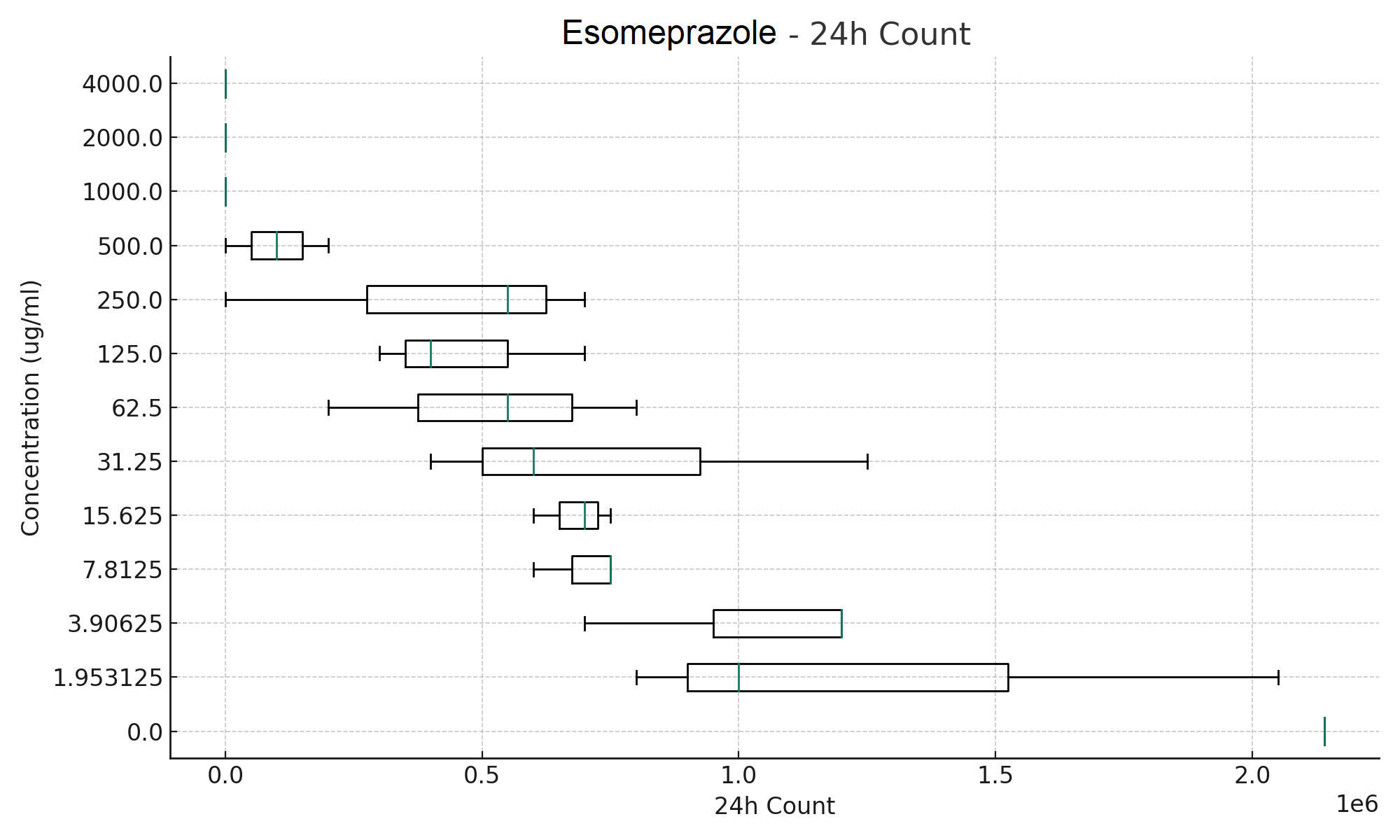

Supplement: Supplementary file 1 — Supplementary Material 1 [file 41598_2025_10668_MOESM1_ESM.zip › Supplementary Figure S4.png]

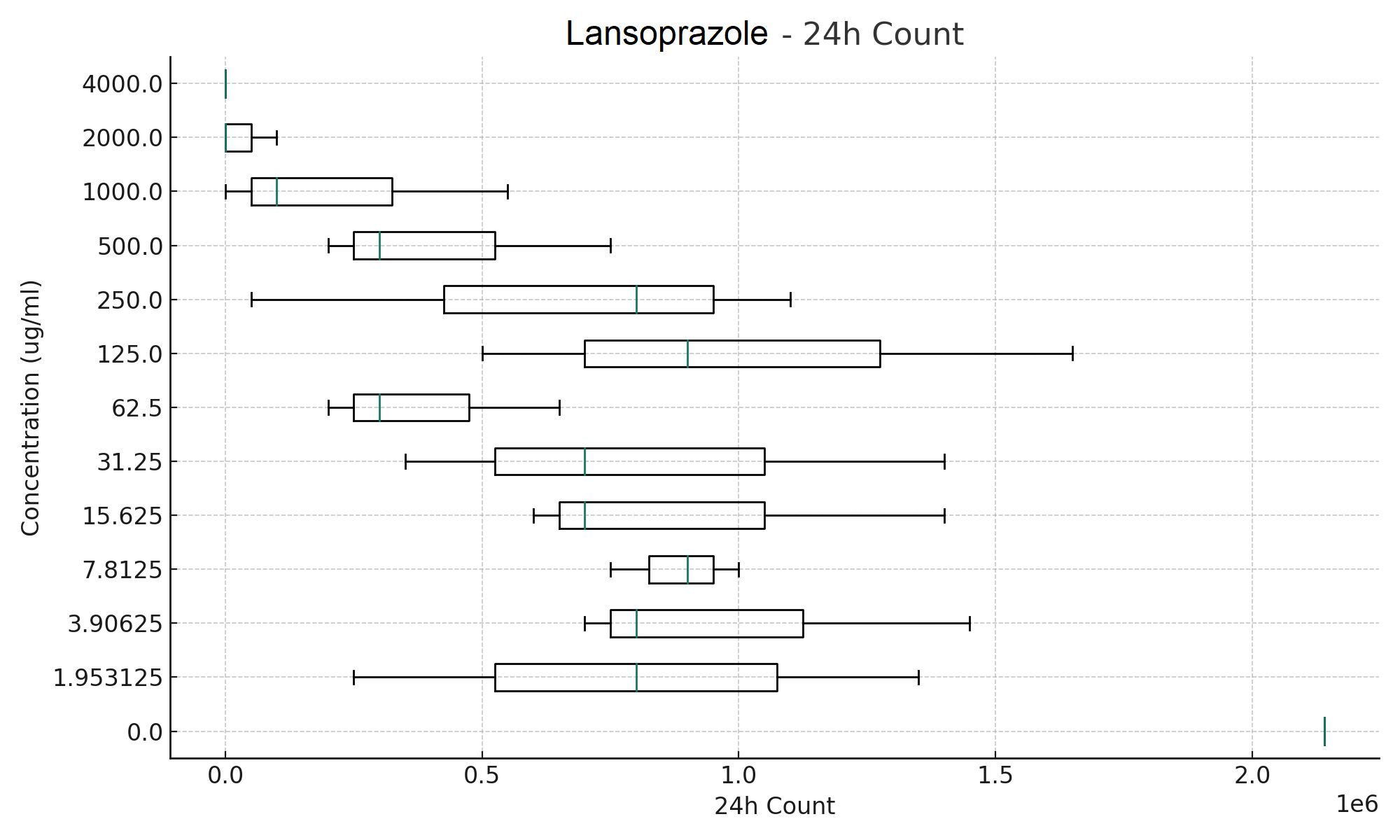

Supplement: Supplementary file 1 — Supplementary Material 1 [file 41598_2025_10668_MOESM1_ESM.zip › Supplementary Figure S5.png]

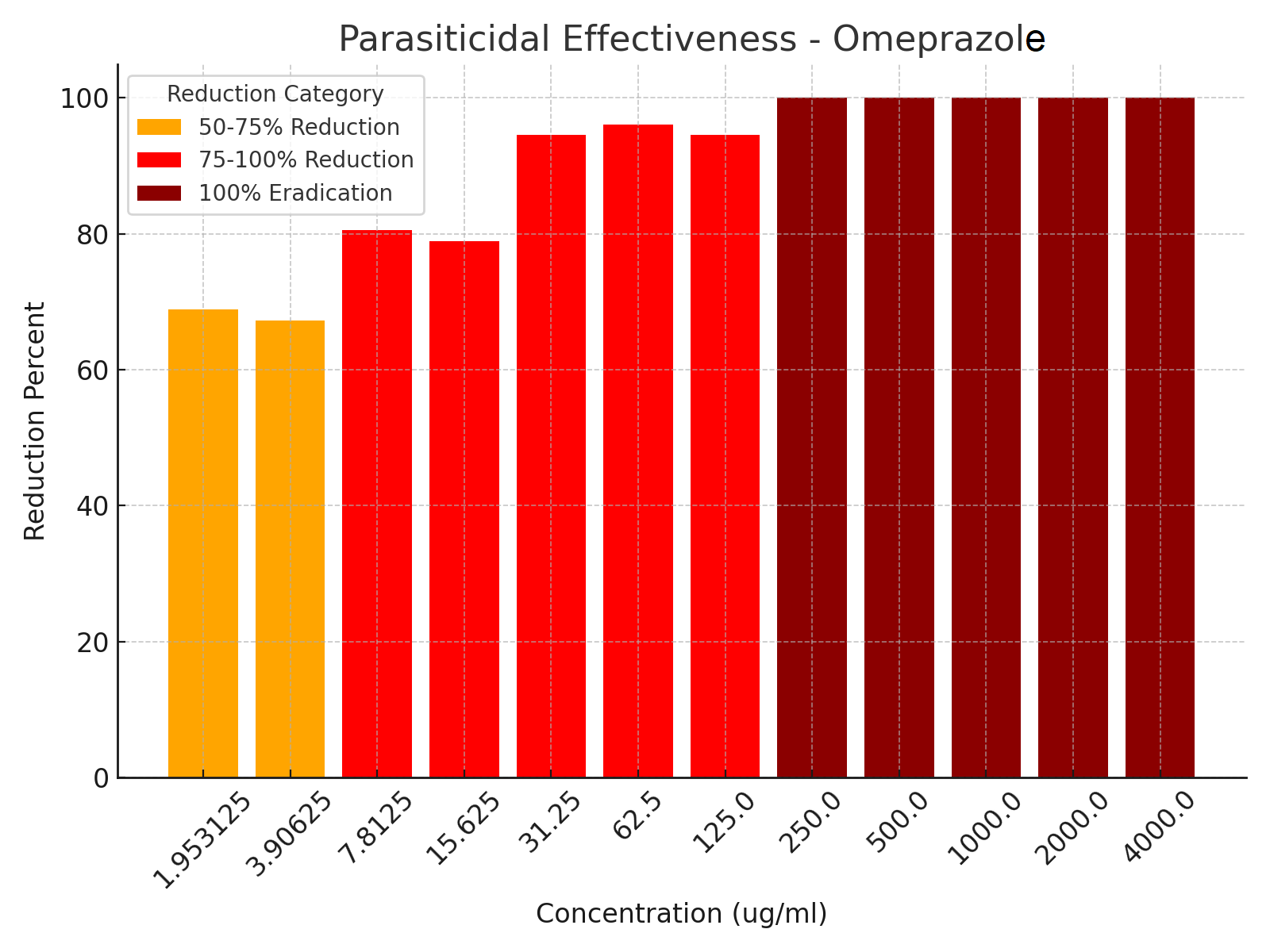

Supplement: Supplementary file 1 — Supplementary Material 1 [file 41598_2025_10668_MOESM1_ESM.zip › Supplementary Figure S6.png]

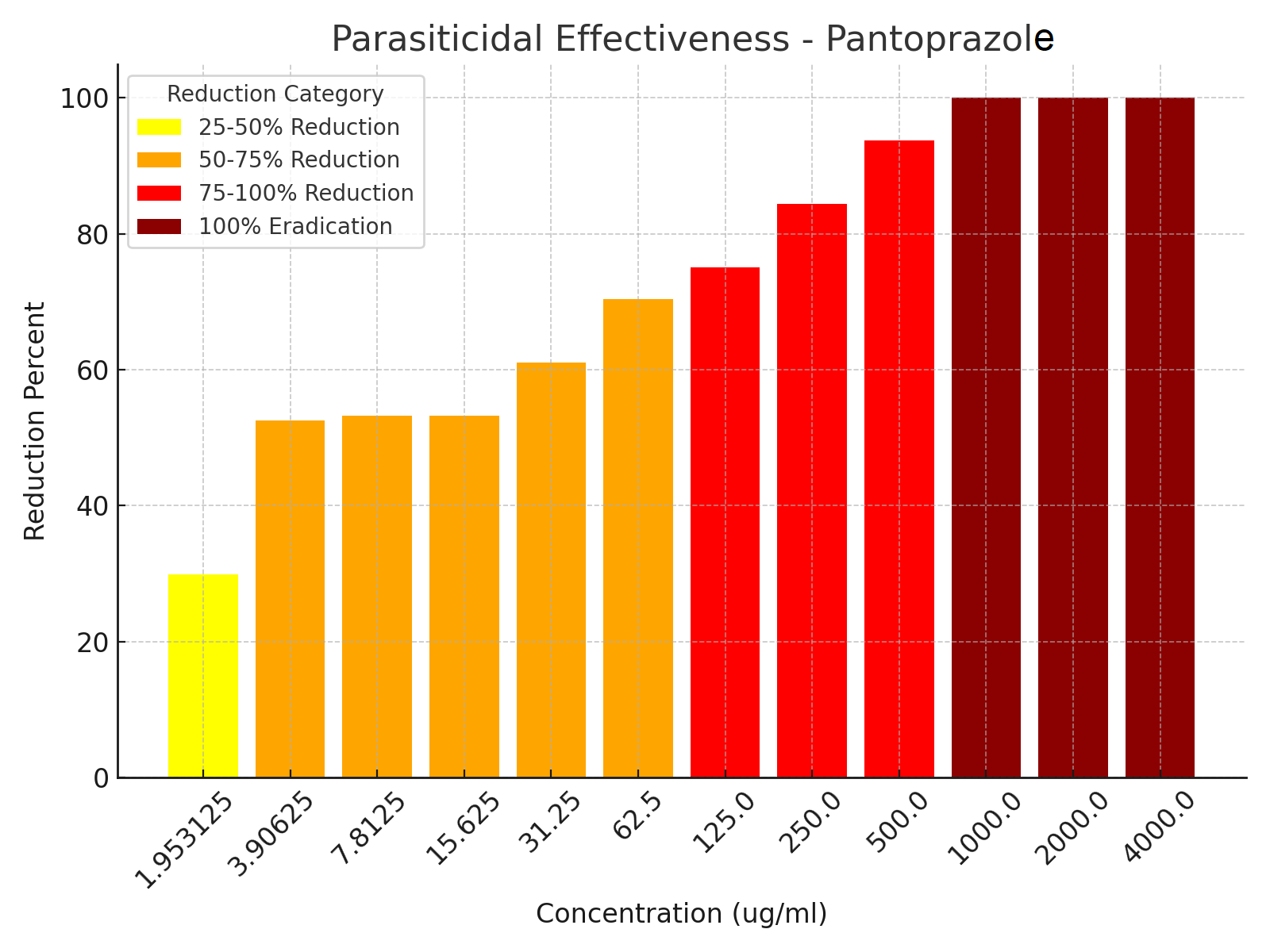

Supplement: Supplementary file 1 — Supplementary Material 1 [file 41598_2025_10668_MOESM1_ESM.zip › Supplementary Figure S7.png]

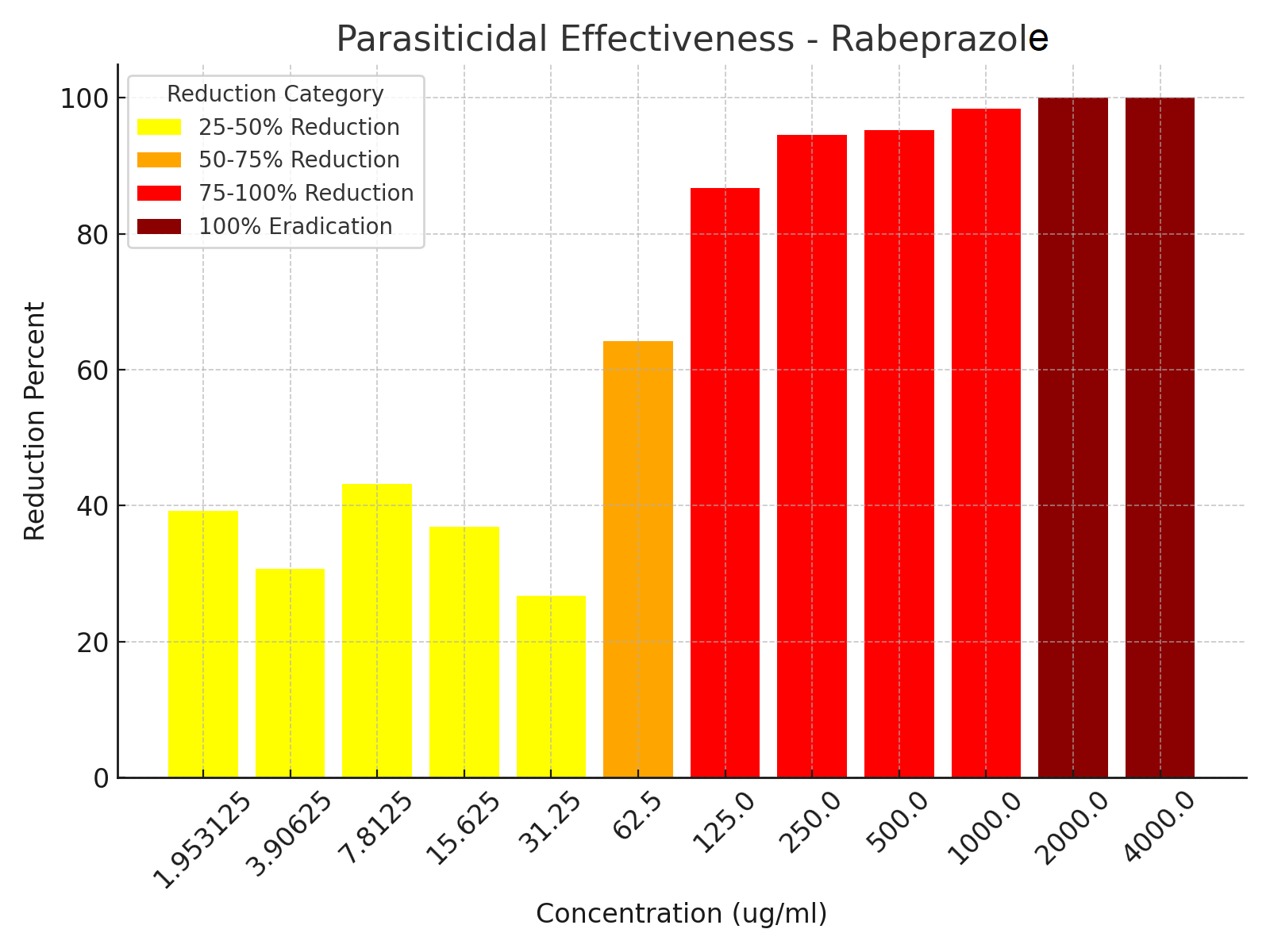

Supplement: Supplementary file 1 — Supplementary Material 1 [file 41598_2025_10668_MOESM1_ESM.zip › Supplementary Figure S8.png]

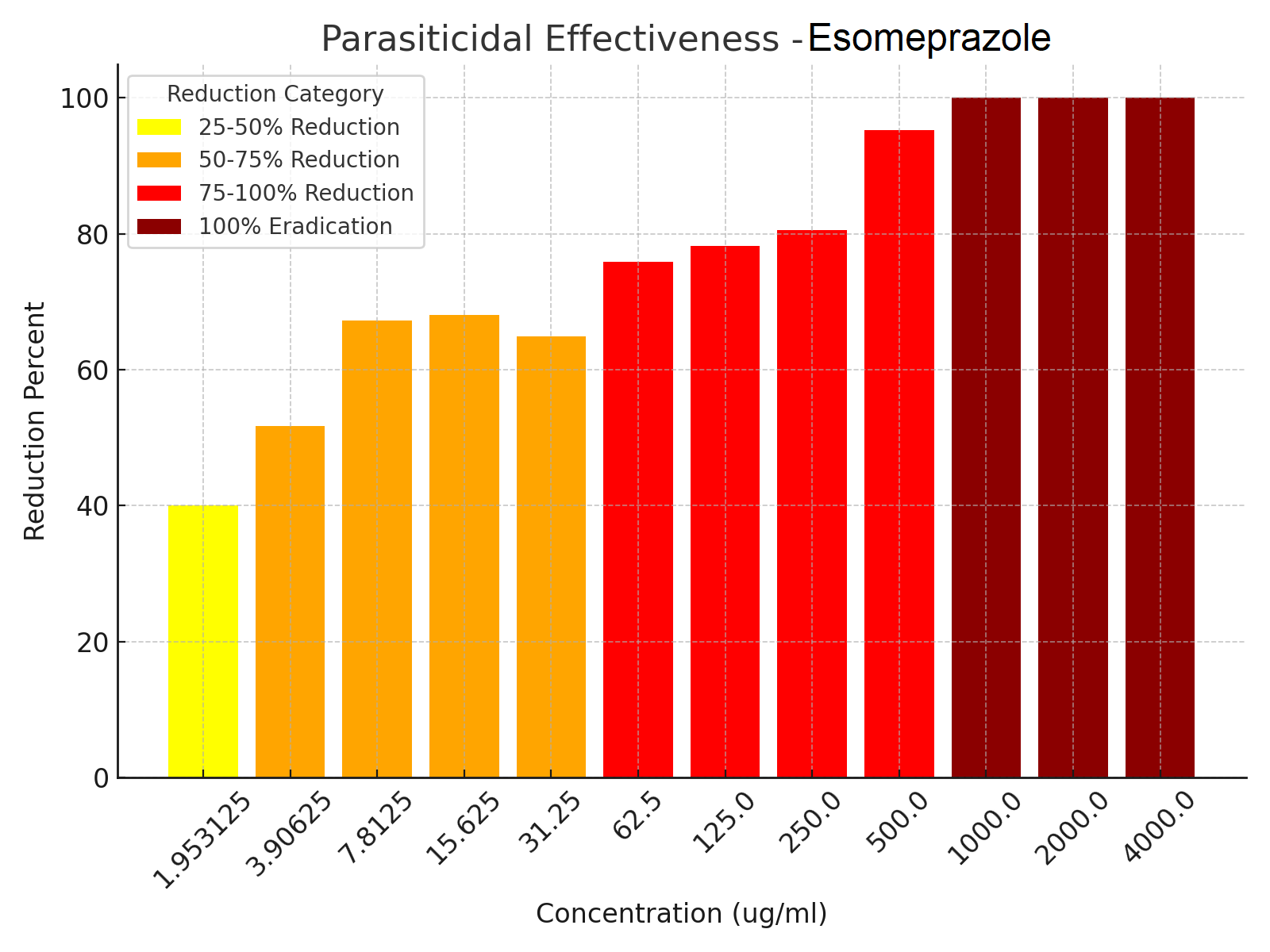

Supplement: Supplementary file 1 — Supplementary Material 1 [file 41598_2025_10668_MOESM1_ESM.zip › Supplementary Figure S9.png]
